# Syntheses and Functional Properties of Phthalocyanines

**DOI:** 10.3390/ma2031127

**Published:** 2009-08-28

**Authors:** Keiichi Sakamoto, Eiko Ohno-Okumura

**Affiliations:** 1 Department of Sustainable Engineering, College of Industrial Technology, Nihon University, 1-2-1 Izumi-cho, Narashino-shi, Chiba-ken 275-8575 Japan; 2 Department of Applied Molecular Chemistry, College of Industrial Technology, Nihon University, 1-2-1 Izumi-cho, Narashino-shi, Chiba-ken 275-8575 Japan; E-Mail: eiko.okumura@tokyokasei.co.jp; 3 Research Institute of Chemical Science, Technology and Education, 8-37-1-104 Narashinodai, Funabashi-shi Chiba-ken 274-0063 Japan

**Keywords:** phtalocyanine, subphthalocyanine, electrochemistry, near infrared absorption

## Abstract

Metal phthalocyanine tetrasulfonic acids, metal phthalocyanine octacarboxylic acids, metal octakis(hexyloxymethyl)phthalocyanines, and metal anthraquinocyanines have been synthesized. Then, zinc bis(1,4-didecylbenzo)-bis(3,4-pyrido)porphyrazines, the cyclotetramerization products of a 1:1 mixture of 3,6-didecylphthalonitrile and 3,4-dicyanopyridine, were synthesized. Futher, subphthalocyanine and its derivatives, with substituents such as thiobutyl and thiophenyl moieties were synthesized. Electrochemical measurements were performed on the abovementioned phthalocyanine derivatives and analogues in order to examine their electron transfer abilities and electrochemical reaction mechanisms in an organic solvent. Moreover, 1,4,8,11,15,18,22,25-octakis(thio-phenylmethyl)phthalocyanes were synthesized. The Q-bands of the latter compounds appeared in the near-infrared region. Furthermore, non-colored transparent films in the visible region can be produced.

## 1. Introduction

**Figure 1:**
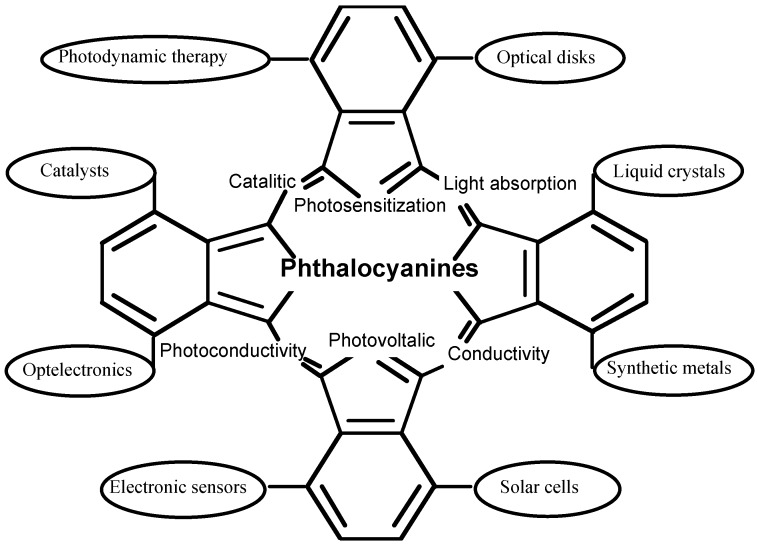
Typical function of phthalocyanine derivatives.

Phthalocyanine derivatives, which have a similar structure to porphyrin, have been utilized in important functional materials in many fields. Their useful properties are attributed to their efficient electron transfer abilities. The central cavity of phthalocyanines is known to be capable of accomodating 63 different elemental ions, including hydrogens (metal-free phthalocyanine, H_2_-PC). A phthalocyanine containing one or two metal ions is called a metal phthalocyanine (M-PC). In the last decade, as a result of their high electron transfer abilities, M-PCs have been utilized in many fields such as molecular electronics, optelectronics, photonics, etc [1,2,3,4,5,6] (Figure 1). The functions of M-PCs are almost universally based on electron transfer reactions because of the 18 π electron conjugated ring system found in their molecular structure.

Further, particular derivatives are known to have potential as second-generation photosensitisers for photodynamic therapy (PDT) of cancer [7] because they show strong absorption of the far-red light between the wavelengths of 600 and 850 nm, which has greater tissue penetration properties [8], and satisfactory photosensitization of singlet oxygen [9].

For some applications, the lower solubility of unsubstituted M-PCs can present problems, but low solubility in common organic solvents can be overcome by the introduction of appropriate substituents onto the ring system. In this context M-PC analogues containing a pyridine (Py) ring in place of one or more of the benzenoid rings are particularly interesting compounds because quaternization of the pyridine nitrogen is expected to confer solubility in aqueous media.

Tetrapyridoporphyrazine M-PC analogues in which all four benzenoid rings are replaced by pyridinoid rings were first synthesized by Linstead and his co-workers in 1937 [10]. They obtained an insoluble product from the self-condensation of 3,4-dicyanopyridine which was presumably a mixture of ‘positional isomers’ or regioisomers. Subsequently, Yokote and Shibamiya reported the synthesis and dying properties of some unsubstituted benzopyridopyridoporphyrazines [11,12] and the ring system attracted the attention of other groups, resulting in a substantial increase in the number of known derivatives [13,14]. Yokote and Shibamiya also reported the synthesis of unsubstituted benzopyridoporphyrazines containing a mixture of benzenoid and pyridinoid rings by cross cyclotetramerization of phthalic anhydride and pyridine carboxylic anhydride [15].

For many applications the absorption maxima of M-PCs are best if moved near the infrared region. The strongest absorption of M-PCs in the visible region, the so-called Q band, can be attributed to the allowed highest occupied molecular orbital (HOMO)—lowest unoccupied molecular orbital (LUMO) (π−π^*^) transition. The Q-band of M-PCs can be moved by bathochromic effects through extension of the π conjugation system such as seen in naphthalocyanines and anthracyanines, but yields of naphthalocyanines and anthracyanines are however usually low. To solve the problem, novel M-PCs having non-peripheral *S*-aryl substituents have been synthesized. These novel M-PCs will show a high strain structure and no liquid crystal properties.

The authors reported a charge transfer thin film exhibiting organic electroluminescent (EL) properties that was made from an organic varnish including an aniline oligomer as a charge transfer material. Cu-PC is used as a standard charge transfer material in the field of organic EL; it is coated on a surface as a thin film using a suitable dry process such as a vacuum method because of its low solubility. There is a need for Cu-PCs having high heat and light resistance for use in wet processes such as spin-coating, spraying, and ink-jet methods because of the demand in large EL display manufacture. Cu-PCs presents the disadvantage of lower transmittance in the visible region because of their strong absorption maxima Q bands.

In the present paper, we describe the synthesis and electrochemical characteristics of some soluble M-PC derivatives and analogues which are expected to be useful as sensitizers for PDT, in photovoltaic cells and laser printing systems. Syntheses of soluble M-PCs [16], including phthalocyanine-4,4’,4’’,4’’’-tetrasulfonic acids **1** having sulfonic acid groups, phthalocyanine-2,3,9,10,16,17,23,24-octacarboxylic acids **2**, 2,3,9,10,16,17,23,24-octakis-(hexoxymethyl)phthalo-cyanines **3** and a novel M-PC derivative, anthraquinocyanine (**4**), which has four 9,10-anthraquinone units in the molecule, have been reported [16,17]. The authors have measured cyclic voltammograms (CVs) and performed chronocoulometric analyses of **1**-**4** in order to estimate their electron transfer properties and the corresponding mechanisms.

Next, we have reported the preparation and characterization of zinc alkybenzopyridoporphyrazines **5**, which were synthesized by reaction of 3,6-didecylphthalonitrile [17,19] and 3,4-dicyanopyridine (or 2,3-dicyanopyridine) [18] in mole ratios of 4:0, 3:1, 1:1, 1:3 and 0:4, respectively. These compounds showed liquid crystal behavior, but incorporation of zinc into the macrocycle inhibited mesophase formation. The 1:1 mole ratio cross cyclotetramerization product has been separated, as the corresponding zinc derivative, with particular attention given to the isolation of the five possible isomers of the zinc bis(1,4-didecylbenzo)-bis(3,4-pyrido)porphyrazine (**5c**). Then zinc bis(1,4-didecylbenzo)-bis(2,3-pyrido)porphyrazine (**5f**) was also synthesized. The compounds **5c** and **5f** exhibit solubility in organic solvents and are expected to have a higher tumor affinity than water soluble M-PCs such as the tetrasulfophthalocyanines. Amphiphilic phthalocyanine derivatives are considered the best compounds for a new generation of photosensitizers for PDT [19] and quaternization of the Py nitrogen in zinc bis(1,4-didecylbenzo)bis(3,4-pyrido)porphyrazine is expected to confer solubility in an aqueous media [15], and lead to good bioavailability and *in vivo* distribution, thus the quaternization of **5c** and **5f** would provide amphiphilic M-PCs.

In general, M-PCs have four isoindole units and a central metal. A class of M-PC-related compounds, the subphthalocyanine (SubPC) derivatives **6**, are the lowest homologues, consisting of three isoindole units and a central boron. Unsubstituted M-PC derivatives are known to have poor solubility in common organic solvents, whereas SubPC derivatives have excellent solubility since these phenomena are attributed to their non-planar crystal structures [7,22,23].

The SubPC derivatives have previously only been used as reagents for ring enlargement reactions leading to unsymmetric M-PCs. Now, another application of SubPC has been recently reported [24,25]. In order to develop new applications of SubPC, we synthesized SubPC and six of its derivatives: tris-(*tert*-butyl)subphtalocyaine (*tert*-butylSubPC; **6b**), dodecylfluorosubphthalocyanine (SubPCF_12_; **6c**), hexakis(*S*-butyl)hexafluoro-subphthalocyanine (SubPC(*S*-butyl)_6_F_6_; **6d**), hexakis(*S*-phenyl)-hexa-fluorosubphthalocyanine (SubPC(*S*-phenyl)_6_F_6_; **6e**), dodecylkis(S-butyl)subphthalo-cyanine (SubPC(*S*-butyl)_12_; **6f**) and dodecylkis(*S*-phenyl)subphthalocyanine (SubPC(*S*-phenyl)_12_; **6g**).

Then, to develop new charge transfer materials, we attempted the synthesis of the 1,4,8,11,15,18,22,25-octakis(thiophenyl)phthalocyanines **15**, novel non-peripheral *S*-aryl substituted M-PCs with linking through sulfur atom electron-donating groups which show near-infrared absorption, high strain structures, no liquid crystal properties and no isomers.

## 2. Results and Discussion

### 2.1. Synthesis of phthalocyanines

**Scheme 1:**
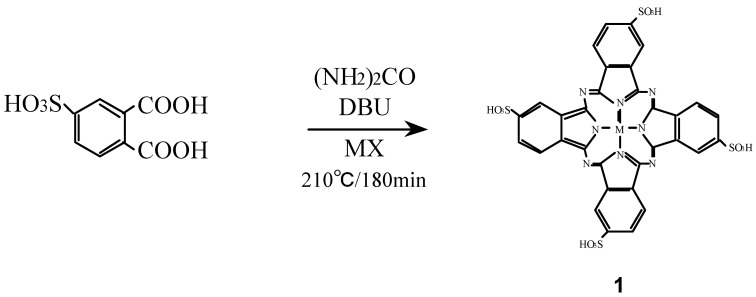
Synthetic pathway to compounds **1**.

Compounds **1** were synthesized from 4-sulfophthalic acid, a metal halide, urea and 1,8-diazabicyclo-[5,4,0]undec-7-ene (DBU) as a catalyst (Scheme 1) [16,26,27].

The position of the absorption peaks in their infrared (IR) and ultraviolet-visible (UV-Vis) spectra, and elemental analytical data of **1** were all in agreement with the proposed structures and literature data [19,24].

**Scheme 2:**
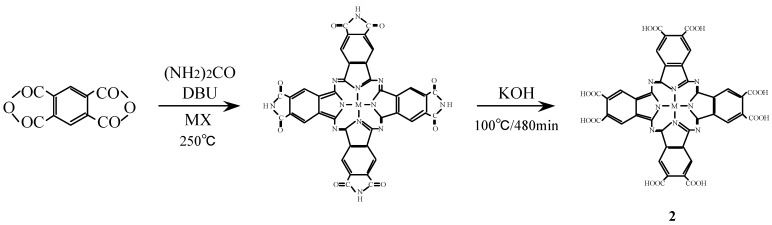
Synthetic pathway to compounds **2**.

Compounds **2** were synthesized from benzene-1,2,4,5-tetracarboxylic dianhydride (pyromellitic dianhydride), a metal halide and urea under the reaction conditions used for the monomer preparation (Scheme 2) [16,26,27]. The IR spectrum of cobalt octasubstituted phthalocyanine synthesized from pyromellitic dianhydride gave a characteristic imide group pattern in the region from 1,600–1,800 cm^-1^. The imide functional groups in the synthesized metal octasubstituted phthalocyanine were changed to carboxylic acida by hydrolysis with potassium hydroxide (KOH). The position of the absorption peaks in the IR and UV-Vis spectra and elemental analysis data of **2** are in agreement with the proposed structure and literature data [28,29].

**Scheme 3:**
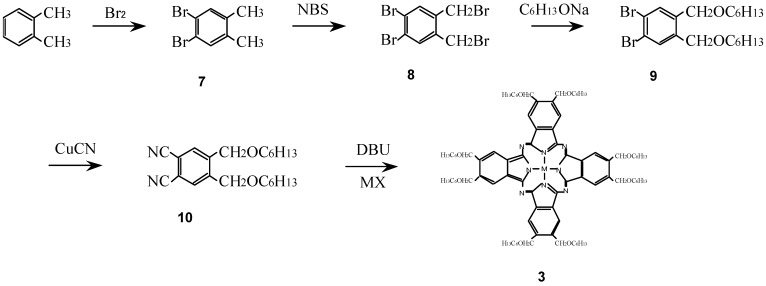
Synthetic pathway to compounds **3**.

Compounds **3** were synthesized from a metal halide and 1,2-dicyano-4,5-bis(hexoxy-methyl)benzene (**10**) which was synthesized in turn from *o*-xylene via 1,2-dibromo-4,5-dimethyl-benzene (**7**), 1,2-dibromo-4,5-bis(bromomethylbenzene) (**8**) and 1,2-dibromo-4,5-bis(hexoxymethyl)-benzene (**9**) (Scheme 3) [16,25,27].

**Scheme 4:**
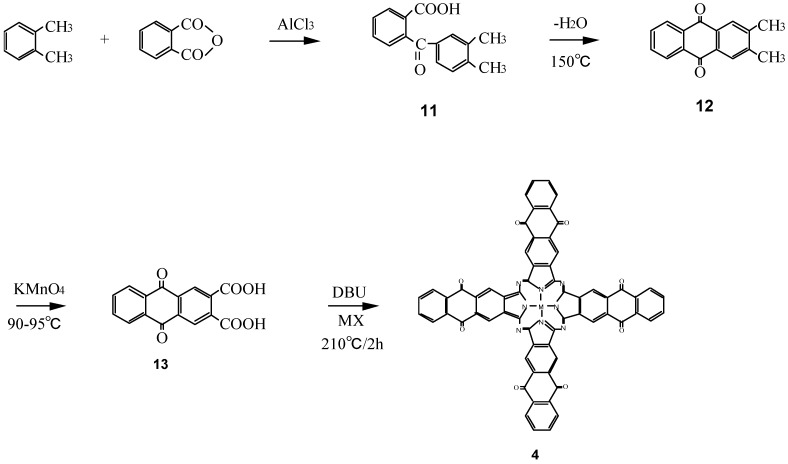
Synthetic pathway to compounds **4**.

Compounds **4** are a new type of phthalocyanine derivative which was synthesized from 9,10-anthraquinone-2,3-dicarboxylic acid (**13**), which in turn was prepared from *o*-xylene and phthalic anhydride via *o*-(3,4-dimethybenzoyl)benzoic acid (**11**), 2,3-dimethyl-9,10-anthraquinone (**12**), and metal halide with urea (Scheme 4) [17]. The intermediates **11** - **13** of **4** have been analyzed by ^1^H-NMR and IR spectra, and elemental analysis and the analytical data were in good agreement with the proposed structures.

The IR spectra of **4** are similar to those of M-PC derivatives which have heterocyclic rings in the molecule and possess characteristic absorption bands. The formation of **4** was confirmed by the appearance of a pyrrole peak and the product’s IR spectrum and the electron absorption band appearing at 680 nm were consistent with the formation of the anthraquinocyanine ring. Yields of **4-M** (M = cobalt, iron and zinc) were largely independent of the kind of central metal used.

The Pariser-Parr-Pople (PPP) self consistent field molecular orbital (MO) method with configuration interaction was employed to calculate the properties of the metal phthalocyanine derivatives synthesized in this work. The PPP-MO calculation was carried out with the programmed software written by Tokita and his co-workers [31].

The calculated electron absorption spectra are shown in Table 1. The Δλ_obs.-calc._ shows the difference between the observed absorption peak λ_max_ value in the Q band which could be attributed to the allowed π - π^*^ transition, and the calculated one. The value of Δλ_obs.-calc._ is in the range of 18–21 nm. The difference in value between the observed and calculated absorption peaks is attributable to the lack of estimation of the central metal in the software.

**Table 1:** The calculated absorption spectra from PPP molecular orbital method.

Compound	Calculated absorption spectra /nm						Δλ obs.-calcs. in Q band / nm
**1**	328	345	394	414	702	809	18
**3**	324	430	385	412	686	802	21
**4**	322	347	395	414	708	803	20

It is confirmed from the PPP-MO calculation that the eight alkoxyalkyl groups in metal octakis(hexoxymethyl)phthalocyanines act as electron-donating substituents, while the eight carbonyl groups in **11** act as electron-withdrawing substituents. Though the PPP-MO method is unable to estimate the effect of the central metal, it is considered that since **3** and **4** were synthesized by this method, the difference between the observed value and the calculated one was within 20 nm, even in the case of **1** and **2**. These substituents produce a red shift of the absorption peak in comparison with unsubstituted M-PC.

Cobalt-**1**, **2**, **3** and **4** have the strongest absorption peaks, around the 680 nm region. These strongest absorption peaks are assigned to the Q band, which could be attributed to the allowed π − π∗ transition [26,32]. The Q band absorption of soluble metal phthalocyanine derivatives synthesized in this work was shifted by 50-80 nm to a longer wavelength in comparison with unsubstituted metal phthalocyanines which appeared around 600 nm. The shift of absorption maxima depends upon the change in electron distribution in the phthalocyanine macrocycle by substituents (Table 2).

**Table 2:** Absorption maximum of Q band in UV-Vis spectrum of cobalt phthalocyanine derivatives.

Compound	λmax in Q band / nm
**1**	649
**2**	684
**3**	665
**4**	688

**Scheme 5:**
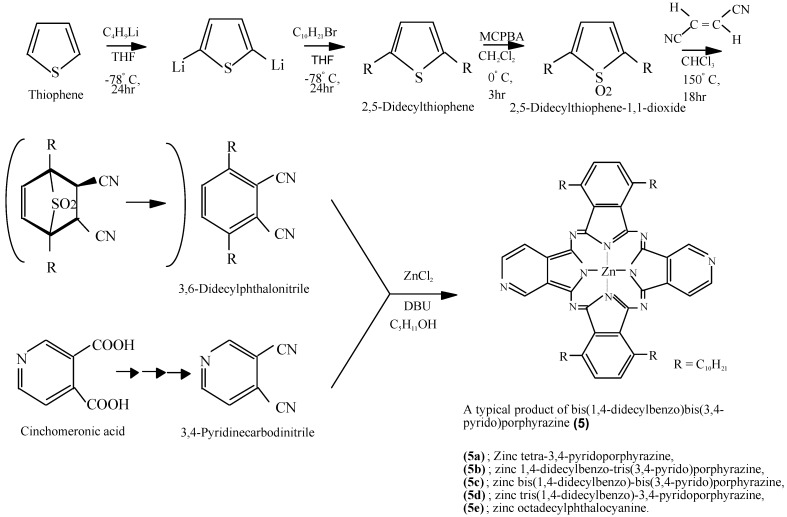
Synthetic pathway to compounds **5**.

Compounds **5** were synthesized by a multi step process. One raw material, 3,6-didecylphthalonitrile, was synthesized from thiophene in three steps via 2,5-didecylthiophene and 2,5-didecylthiophene-1,1-dioxide. The other raw material, 3,4-pyridine carbodinitrile, was synthesized from cinchomeronic acid in three steps (Scheme 5) [33]. Compound **5f** was synthesized from 2,5-didecylthiophene-1,1-dioxide and 2,3-pyridinecarbodinitrile, which prepared from quinolinic acid [34].

The intermediates of **5** were analyzed by IR, ^1^H-NMR, mass (MS) spectrometry and elemental analysis. The analytical data for all compounds were in good agreement with the proposed structures. Compounds **5** displayed strong absorption peaks around 680 nm for the Q band [18,20,33,34,35,36]. The Q band absorption of **5** shifted by 50-80 nm to a longer wavelength in comparison with unsubstituted phthalocyanines (Table 3).

**Figure 2:**
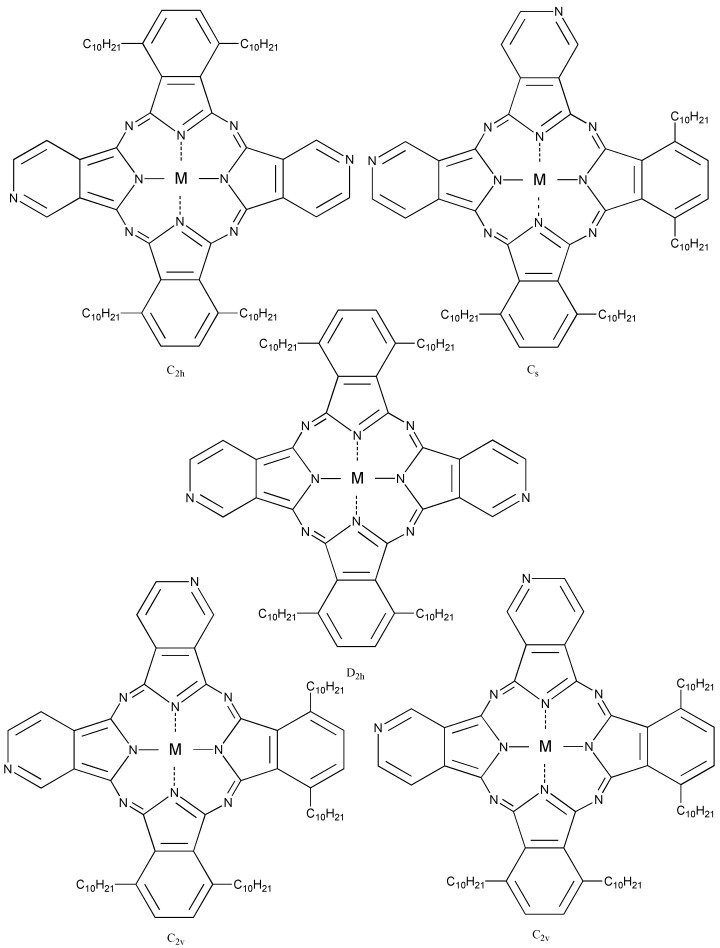
Regioisomers of metal bis(1,4-didecylbenzo)-bis(3,4-pyrido)porphyrazine **5c**.

Compound **5c**, synthesized from a 1:1 mol ratio of raw materials, has five regioisomers (Figure 2). It has two non-peripheral substituted benzenoid and pyridinoid rings, located at different positions. In the molecule the pyridinoid rings can be adjacent or opposite to each other. In the case of adjacent pyridinoid rings, three types of isomer exist, depending on the orientation of the ring. Meanwhile, two types of isomers exist in the case of opposite pyridinoid rings. We attempted to separate the regioisomers of the compound **5c** using thin layer chromatography (TLC) (Merck silica gel 60F_254_ on aluminum sheet, eluent: toluene - Py, 7:3). Four green- to blue-colored fractions presenting different ^1^H-NMR, UV-Vis and fluorescent spectra were obtained in 26.1, 17.4, 17.4 and 39.1% yield, respectively, and can be attributed to four of five possible regioisomers of the **5c**. These fractions were numbered from 1, 2, 3 and 4, according to the R_f_ values (0.95, 0.91, 0.75 and 0.65, respectively). Each fraction was recovered by scraping the absorbent from the TLC plate, extracting with Py, filtering and removing the solvent.

**Table 3:** The Q band maxima of compounds **5** after purification by TLC.

Compound	Q band λmax toluene /nm		
**5a**	675*	664*	603*
**5b**	686	635	617
**5c**	686	636	717
**5d**	686	635	617
**5e**	703	634	

* in pyridine.

The regioisomers of the **5c** were estimated by ^1^H-NMR, UV-Vis Q band absorption and MO calculations. The MO calculations of the isomers were performed using the ZINDO/S semi-empirical CI configurations available in the HyperChem 5.1Pro software package in order to obtain their theoretical Q band absorptions.

**Scheme 6:**
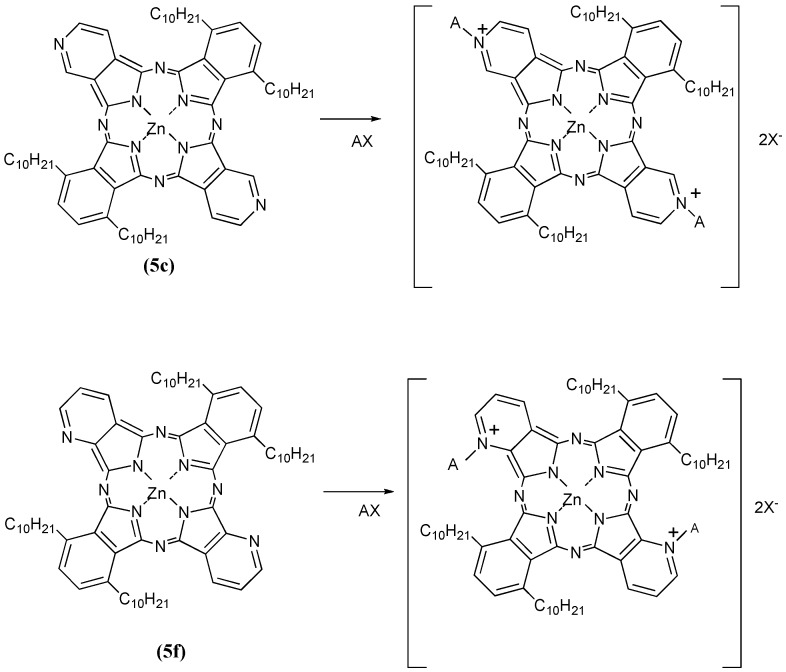
Quaternization reactions of compounds **5c** and **5f** with quaternizing agents (AX).

Compounds **5c** and **5f** were next further reacted with quaternizing agents (AX) such as monochloroacetic acid (MCAA), diethyl sulfate (DES) and dimethyl sulfate (DMS) in *N,N*-dimethylformamide (DMF) as solvent at 140 °C (Scheme 6).

Yields of products of **5c** using MCAA, DES and DMS as quaternizing agents were 23%, 17% and 28%, respectively. The corresponding yields of products with the same reagents when **5f** was the substrate were 24%, 21% and 25%, respectively. After reaction with the quaternizing agents, the products of **5c** and **5f** were identified through spectroscopic techniques such as ^1^H-NMR, IR and UV-Vis spectrometry. When the quaternizing agent was MCAA or DMS, it is confirmed that *N*-CH_2_COOH or *N*-CH_3_ bonds were obtained on the pyridinoid rings, while when DES was used, we noted that quaternization was not achieved but rather sulfonation occurred. The shapes of the UV-Vis spectra in Py solution changed after quaternatization, and it is thought that interactions between the molecules are complicated when DES and DMS were used as quaternizing agents, since the quaternized products easily aggregate in aqueous media. After reaction with quaternizing agents, all compounds became water soluble, and acquired amphiphilic properties (Table 4).

**Table 4:** Solubilities of **5c** and quaternized compounds with MCAA, DES and DMS.

Compound	Toluene	Chloroform	Pyridine	Water
**5c**	Soluble	Soluble	Soluble	Insoluble
With MCAA	Soluble	Soluble	Soluble	Soluble
With DES	Soluble	Soluble	Soluble	Soluble
With DMS	Soluble	Soluble	Soluble	Soluble

**Figure 3:**
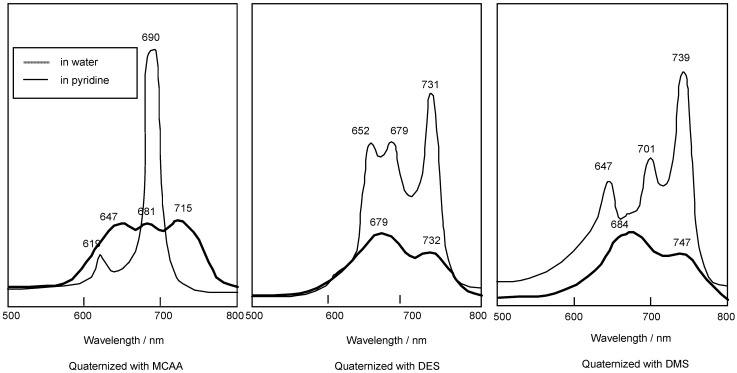
UV-Vis spectra of **5c** quaternized with MCAA, DES and DMS. The UV-Vis spectra of each quaternized compound was measured in pyridine solution or water.

Compounds **5c** and **5f** are readily soluble in Py, with their strongest absorption appearing at 687 nm (log ε_max_ 4.81). The Q band absorption of the compound quaternized with DMS showed a peak at 739 nm, shifted by 56 nm to longer wavelength in comparison with compounds reacted with MCAA and DES. After reacting with MCAA, DES and DMS, all compounds are very soluble in water, and they show strongest absorptions in the Q band at 715, 731 and 747 nm, respectively (Figure 3).

**Figure 4:**
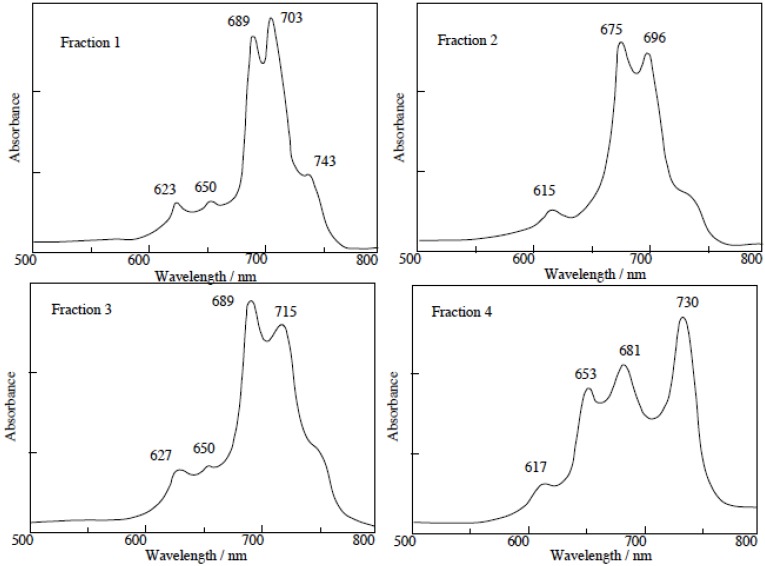
UV-Vis spectra of each regioisomer in **5c** quaternized with DMS. The UV-Vis spectra of each quaternized compound was measured in Py solution.

The separated regioisomers of **5c** were also quaternized with DMS. Yields of products were 11%, 9%, 15% and 11% for fractions 1, 2, 3 and 4, respectively. Quaternized regioisomers were also identified using spectroscopic techniques such as ^1^H-NMR, IR, UV-Vis and fluorescence spectra. A comparison of the UV-Vis spectra before and after the quaternization of the regioisomers with DMS, showed that the Q band absorptions of quaternized regioisomers were split into two or more bands. After quaternization, the Q band absorptions were also moved to longer wavelengths (Figure 4).

In general, fluorescence spectra appear as the mirror images of excitation spectra. Excitation spectra show the same absorption band profiles. Fluorescence spectra show longer wavelengths than absorption and excitation spectra. For the quaternized products, except for fractions 1 and 4, the fluorescence spectra showed longer wavelengths than their corresponding absorption spectra.

In fractions 1 and 4, a degree of overlap between absorption and fluorescence spectra was observed. These phenomena result from fluorescence re-absorption or re-emission. Accordingly, the fluorescence spectra of fractions 1 and 4 showed shorter wavelength than the longest wavelength of the absorption bands.

**Scheme 7:**
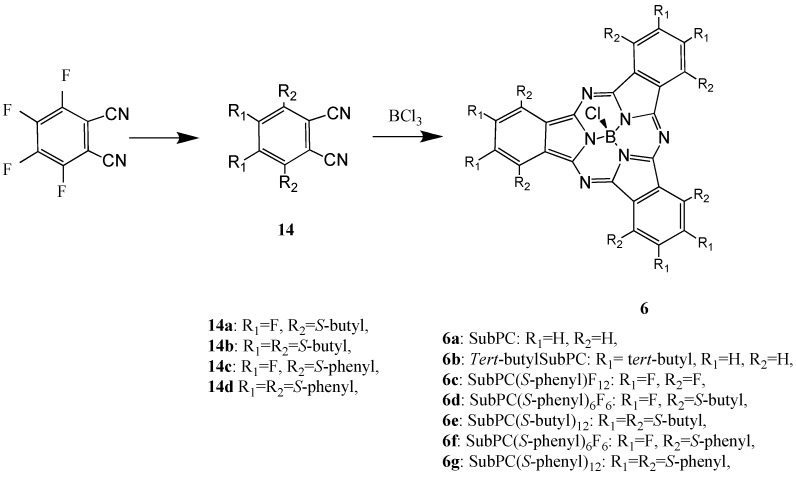
Synthetic pathway to compounds **6**.

Compound **6a** was synthesized from 1,2-dicyanobenzene. Compound **6c** was synthesized from 1,2-dicyano-3,4,5,6-tetrafluorobenzene (Scheme 7) [36]. Compounds **6d-g** were synthesized from the precursors 1,2-dicyano-3,6-bis(thiobutyl)-4,5-difluorobenzene (**14a**), 1,2-dicyano-3,4,5,6-tetrakis(thio-butyl)benzene (**14b**), 1,2-dicyano-3,6-bis(thiophenyl)-4,5-difluorobenzene (**14c**) and 1,2-dicyano-3,4,5,6-tetrakis(thiophenyl)benzene (**14d**), respectively.

Compounds **6d-g** were synthesized from the precursors 1,2-dicyano-3,6-bis(thiobutyl)-4,5-difluoro-benzene (**14a**), 1,2-dicyano-3,4,5,6-tetrakis(thiobutyl)benzene (**14b**), 1,2-dicyano-3,6-bis(thiophenyl)-4,5-difluorobenzene (**14c**) and 1,2-dicyano-3,4,5,6-tetrakis(thiophenyl)benzene (**14d**), respectively. The precursors of the SubPC derivatives were analyzed and the analytical data were in good agreement with the proposed structures.

The target SubPC and derivatives were analyzed by UV-Vis, IR and ^1^H-NMR spectroscopy and the analytical data were in good agreement with the proposed structures. Absorption bands in the IR spectra of SubPC and its derivatives are assigned as follows: peaks around 1,600 cm^-1^ are aromatic ring absorptions; those in the 1,200–1,050 cm^-1^ region are mainly absorption from out-of-plane bending of CH and NH in pyrrole rings, in which cyclotrimerization is found. These absorption bands in the IR spectrum are characteristic peaks of M-PCs.

The absorption maxima of SubPC and its derivatives appeared around 560 - 630 nm in benzene solution. The Q band absorption of SubPC and its derivatives is shifted by around 100 nm to a shorter wavelength in comparison with normal metal phthalocyanine derivatives, in which they appear around 650 nm. The shift of absorption maxima depends upon the change in electron distribution and the size of the macrocycle. Absorption maxima of SubPC derivatives shifted to longer wavelength with increasing molecular weight. The synthesized SubPC and its derivatives were sufficiently soluble in some organic solvents such as dichloromethane, chloroform, acetone and DMF.

**Scheme 8:**
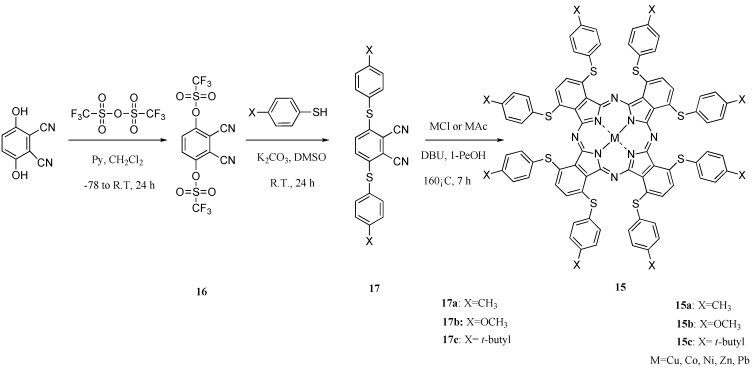
Synthetic pathway to compounds **15**.

The 1,4,8,11,15,18,22,25-octakis(thiophenyl)phthalocyanine compounds **15** were synthesized in three steps via the intermediates phthalonitrile-3,6-ditriflate (**16**) and 3,6-bis(thiophenyl)phthalonitriles (**17**). Intermediate **16** was synthesized from 2,3-dicyanohydroquinon and trifluoromethanesulfonic anhydride for 24 h in accordance with a description from the literature [37]. Intermediates **17** were synthesized, respectively, from **1** and thiophenols such as *p*-toluenethiol, 4-methoxybenzenethiol and tert-butylthiophenol at room temperature for 24 h to obtain 3,6-bis(thiophenylmethyl)phthalonitrile (**17a**), 3,6-bis(thiophenylmethoxy)phthalonitrile (**17b**) and 3,6-bis(thiophenyl tert-butyl)phthalonitrile (**17c**). Intermediates **16** and **17** were analyzed using IR and ^1^H-NMR spectroscopy, and elemental analysis. Their analytical data showed good agreement with the proposed structures (Scheme 8).

1,4,8,11,15,18,22,25-octakis(Thiophenylmethyl)phthalocyanines (**15a**), 1,4,8,11,15,18,22,25-octa-kis(thiophenylmethoxy)phthalocyanines (**15b**) and 1,4,8,11,15,18,22,25-octakis(thiophenyl *tert*-butyl)phthalocyanines (**15c**) were synthesized, respectively, from the corresponding intermediates **17a**, **17b**, and **17c** and metal salt in 1-pentanol in the presence of DBU as catalyst for 7 h (Scheme 1) [38]. As metal salts, chloride or acetate of copper, cobalt, nickel, zinc, and lead were chosen [39]. Metal-free compounds **15** were obtained directly by refluxing **17** in 1-pentanol. The products were isolated using column chromatography on silica gel with toluene as eluent. The most readily apparent feature of the compounds **15** is their solubility in various solvents. The target compounds **15** were analyzed using elemental analysis and MS spectroscopy. The analytical data showed good agreement with the proposed structures.

### 2.2. Electrochemical Properties

Cyclic voltammetry (CV) is often used in electrochemistry studies. It consists of the cyclic potential of a stationary electrode immersed in a quiescent solution and measuring the resulting current. The excitation signal is a linear potential scan with a triangular waveform. This triangular potential excitation signal sweeps the potential of the working electrode. The triangle returns at the same speed and permits the display of a complete voltammogram. Therefore, if a molecular is reduced in the forward scan, it will be reoxidized on the reverse scan. The current response shows the upper half, cathodic (reduction) and the lower half, an anodic (oxidation) peak.

**Figure 5:**
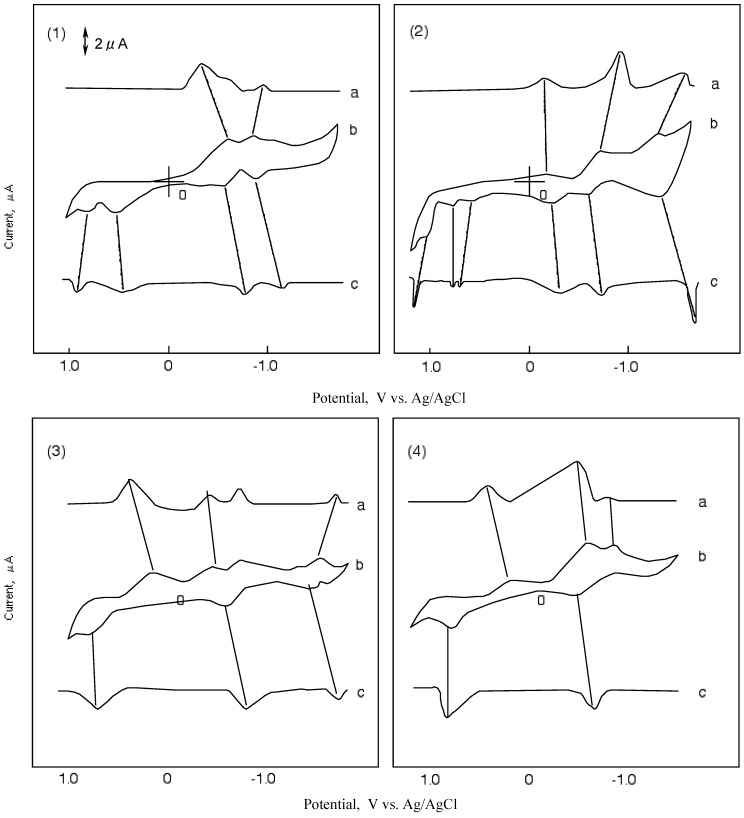
Cyclic voltammograms and their first differential curves of covalt phthalocyanine derivatives in dimethylsulfoxide (DMSO) with 0.1 mol·L^-1^ tetrabutylammonium perchlorate (TBAP), scan rate: 50 mV·s^-1^; a): First differential curve of upper half of cyclic voltammogram; b): Cyclic voltammogram; c): First differential curve of lower half of cyclic voltammmogram; (1) Cobalt phthalocyanine tetrasulfonic acid; (2) Cobalt phthalocyanine octacarboxylic acid; (3) Cobalt octakis(hexoxymethyl)-phthalocyanine; (4) Cobalt anthraquinocyanine.

Figure 5 shows CVs and their first differential curves for compounds **1**, **2**, **3** and **4**, respectively. The reduction and oxidation potentials of cobalt-**1**, **2**, **3** and **4** are summarized in Table 5. The reported potentials are the midpoint potential of anodic and cathodic peaks for each couple, Emid, and the peak potential for the irreversible step.

The CV of cobalt-**1** showed two cathodic peaks at -0.63 and -1.00 V vs. silver (Ag)/ silver chloride (AgCl) saturated sodium chloride, and four anodic peaks at 0.89, 0.67, -0.60 and-0.90 V vs. Ag/AgCl. The peaks of cobalt-**1** are attributed to a five reduction stage. The first reversible reduction potential at -0.62 V vs. Ag/AgCl and the first oxidation potential of cobalt-**1** appeared at 0.67 V vs. Ag/AgCl.

In the case of cobalt-**2**, three cathodic peaks and six anodic peaks appeared. The peaks were sorted into three reversible reduction couples at -0.24, -0.66 and -1.39 V vs. Ag/AgCl, and three irreversible oxidation waves at 0.67, 0.87 and 1.06 V vs. Ag/AgCl. The reduction and oxidation of metal phthalocyanine derivatives are due to the interaction between the phthalocyanine ring and the central metal [40,41]. The reduction and oxidation potential of cobalt phthalocyanine derivatives reported by Rollmann [42] and Orihashi [43] showed more negative values than in the case of cobalt-**1** and **2**. Carboxylic and sulfonic groups are electron-withdrawing groups so they are expected to reduce the electron charge in the phthalocyanine ring. The change in the redox potential is due to the kind and number of substituents.

The CV of cobalt-**3** showed four cathodic peaks at 0.16, -0.49, -0.73 and -1.54 V vs. Ag/AgCl, and three anodic peaks at 0.73, -0.61 and -1.47 V vs. Ag/AgCl. Cobalt-**3** has two irreversible oxidations at 0.16 and 0.73 V vs.Ag/AgCl, and two pairs of reversible reduction potential. Since the hexyloxymethyl substituent is an electron-donating group, the negative charge on the phthalocyanine ring is expected to increase.

The CV of cobalt-**4** showed a unique shape in comparison with the other compounds cobalt-**1**, **2** and **3**. The shape of the CV for cobalt-**4** showed three cathodic peaks at 0.19, -0.69 and -0.95 V vs. Ag/AgCl, and two anodic peaks at 0.87 and -0.58 V vs. Ag/AgCl. Cobalt-**4** has almost one pair of reversible potential.

 The ΔΕ values in Table 5 are the anodic peak to cathodic peak separation located in the oxidation (negative) potential region. The ΔΕ values, except for cobalt-**2**, are around 100 mV and the redox processes are the same for these molecules.

**Table 5:** Redox potentials of cobalt phthalocyanine derivatives.

Compound	Potential / V vs. Ag/AgCl						
	Reduction				Oxidation		
**1**	-1.63*	-0.95	-0.62		0.67*	0.89*	
ΔE**		0.98	0.34				
**2**	-1.39		-0.66	-0.24	0.67*	0.87*	1.06*
ΔE**	1.60		2.27	0.84			
**3**	-1.50	-1.25*	-0.67	-0.49*	0.16*	0.73*	
ΔE**	0.88	1.27					
**4**	-0.95*	-0.63			0.19*	0.87*	
ΔE**		1.07					

Potentials of reversible wave are midpoint of anodic and cathodic praks for each couple, E_1/2_.

*Irreversible peak; ** The anodic peak to cathodic peak separation for reversible couple.

Kadish *et al.* have suggested that the potential difference between the reduction and oxidation is expressed in the HOMO - LUMO energy gap [44]. This potential difference decreases from 1.36 V for cobalt-**2** to 0.65 V for cobalt-**7**. The values of λ_max_ in the Q band shown in Table 5 correlate with the potential difference between the reduction and oxidation of cobalt-**1**, **2**, **3** and **4**.

**Figure 6:**
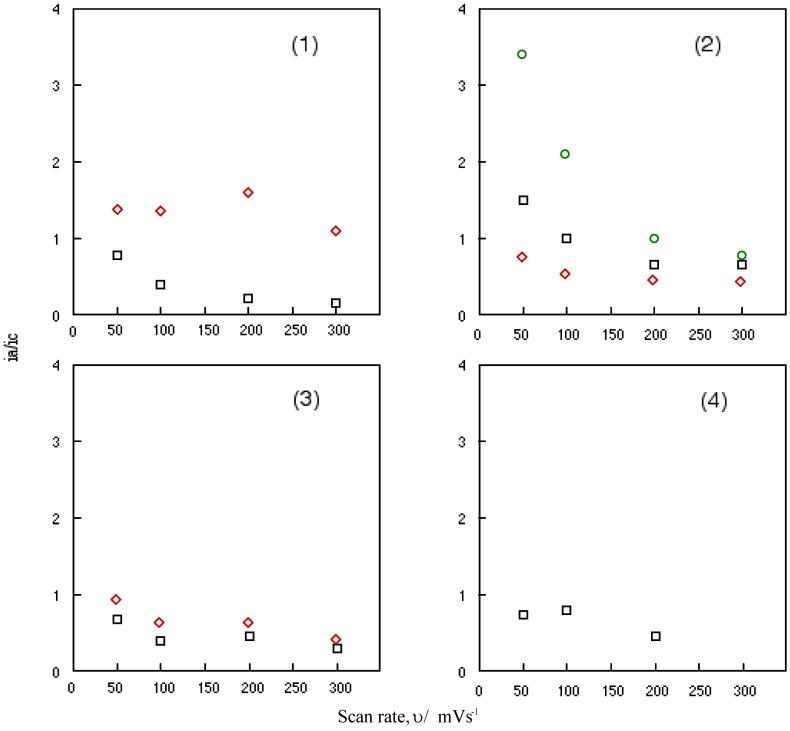
Change in the anodic to cathodic current ratio with scan rate for cobalt phthalocyanine derivatives. (1) Cobalt phthalocyanine tetrasulfonic acid. (2) Cobalt phthalocyanine octacarboxylic acid. (3) Cobalt octakis(hexoxymethyl)phthalocyanine. (4) Cobalt anthraquinocyanine. □: First redox couple. ◇: Second redox couple. ○: Third redox couple.

Figure 6 shows the change in the ratio of anodic peak current to the cathodic current of cobalt-**1**, **2**, **3** and **4** with a scan rate, υ. Nicholson and his co-worker have been studied the relationship between the ratio of anodic to cathodic peak current, i_a_/i_c_ and the scan rate, υ [45]. When the relationship is unity, the system involves a reversible or catalytic reaction. This relationship between the anodic to cathodic peak current ratio of a reversible couple, i_a_/i_c_ and the scan rate, υ serves as a quick test for electrochemical mechanism associated with a preceding or succeeding reversible or irreversible chemical equilibrium. The scan rate varied from 0.05 to 0.3 Vs^-1^ in this work.

It is shown that the ratio of anodic to cathodic peak current, i_a_/i_c_ decreased continuously with an increasing scan rate, υ for all reversible couples of cobalt-**1**, **2**, **3** and **4**. The reversible reduction couples of cobalt-**1**, **2**, **3** and **4** are characterized as a fast reversible electron transfer followed by a reversible chemical reaction. The value of anodic to cathodic peak current ratio, i_a_/i_c_ converges when the ratio is extrapolated to zero of scan rate, υ.

The potentials of anodic to cathodic peak potential ΔΕ are around 100 mV, except for cobalt-**2**. Extrapolated to zero scan rate, υ, the ΔΕ values approach close to 60 mV. These data suggest that the electrode processes of cobalt-**1**, **2**, **3** and **4** take place by almost one-electron transfer. The midpoint potential of cathodic to anodic peak, E_mid_ is independent of scan rate, υ and have constant values. Consequently, it is thought that these electrode processes are complicated diffusion-controlled electron transfers involving some weak adsorption with the oxide of cobalt-**1**, **2**, **3** and **4**.

**Figure 7:**
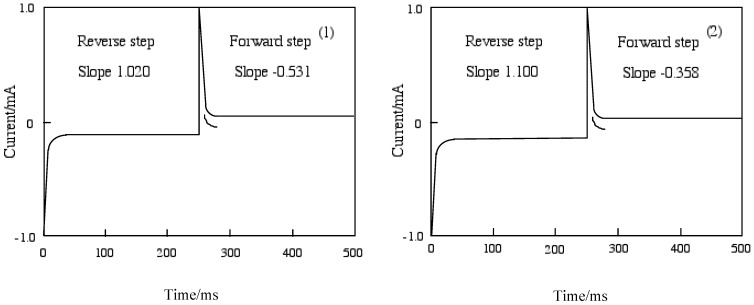
Chronoamperometry and the slope calculated from Cottrell plot of cobalt phthalocyanine tetrasulfonic acid. Potential step; -1.2–1.6 V vs. Ag/AgCl, time interval: 250 ms. (1) Cobalt phthalocyanine tetrasulfonic acid. (2) Cobalt phthalocyanine octacarboxylic acid.

Figure 7 shows chronoamperometry and the slope calculated from Cottrell plots of cobalt-**1** and **2**. The current of the Cottrell plot is a measurement of the rate for electrolysis at the electrode surface. Electrolysis is controlled with a mass transfer by diffusion on the electrode so that the diffusion constant implies the rate of electrolysis. The slope means the diffusion constant in each step, and the forward step indicates the reduction reaction and the reverse step is oxidation. The oxidation processes of cobalt-**1** and **2** are slower than the reduction. The oxidation of cobalt-**1** was lower than cobalt-**2**, while cobalt-**2** was lower reduction ability than cobalt-**1**.

The chronocoulometry was measured by a chronoamperometry treatment in which the current response was integrated to give a response to the charge. The charge-time curve of the forward step for chronocoulometry is the chronoamperometric integral.

The extent of diffusion control increases systematically as the standard potential becomes positive for cobalt-**1** and **2**. In the case of cobalt-**1**, the electron charge was reached at about 30 μC in the forward step and was decreased to 15 μC, and reverse steps were attenuated to 0 μC with 70 ms in the reduction side from -1200 to 0 mV vs. Ag/AgCl potential. In the oxidation side from the 0 to +1,600 mV vs. Ag/AgCl step, chronocoulometry had a linear forward step and a flat reverse step indicating no faradic activity for all compounds. It is thought that reduction and oxidation take place in different pathways.

**Figure 8:**
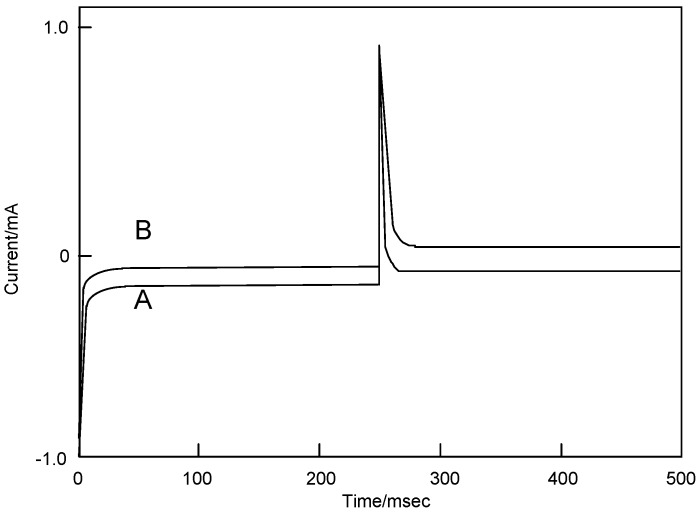
Chronoamperometry of cobalt phthalocyanine tetrasulfonic acid. (A) Potential step; -1.2–0.0 V vs. Ag/AgCl, step width: 250 ms. (B) Potential step; -1.2–1.6 V vs. Ag/AgCl, step width: 250 ms.

Figure 8 shows the chronoamperometry of cobalt-**3** following applied voltage pulse from -1,200 to 0 mV vs. Ag/AgCl and from -1,200 to +1,600 mV vs. Ag/AgCl, and the reversible pulse. Chronoamperometry involves the measurement of the current-time response to a potential step excitation signal. A large cathodic current flows immediately when the potential is stepped up from the initial value, after that it slowly attenuates. The reduction step exhibited the same behavior in comparison with both potential steps.

The current - time curve for chronoamperometry is expressed by the Cottrell Equation (1):

(1) $$i=\frac{{\mathrm{nFACD}}^{1/2}}{\pi^{1/2}t^{1/2}}={\mathrm{Kt}}^{-1/2}$$

where, i: current (A), n: number of electrons transferred per ion or molecule (mol^-1^), F: Faraday’s constant (96,485 C/mol), A: electrode area (2.0·10^-2^ cm^2^), C: concentration (mol/cm^3^), D: diffusion constant (cm/s), t: time (s).

 Electron processes in the systems are the diffusion-controlled electron transfers mentioned above. Relationships between the current and square root of time are considered to respresent a finite diffusion for cobalt-**1**, **2**, **3** and **4**. Table 6 shows the slope of the relationships between the current and square root of time (Cottrell plots). The current of the Cottrell plot is a measure of the rate for electrolysis at the electrode surface. Electrolysis is controlled with a mass transfer by diffusion on the electrode so that the diffusion constant implies the rate of electrolysis. In this table, the slope means the diffusion constant in each step, and the forward step indicates the reduction reaction and the reverse step is oxidation. The oxidation processes of cobalt-**1**, **2**, **3** and **4** are faster than the reduction. The oxidation was decreased in the following order: cobalt-**2**, **1**, **3** and **4**. On the other hand, the reduction decreased in the following order: cobalt-**3**, **1**, **2** and **4**.

**Table 6:** The slope and intercept of Cottrell plot for cobalt phthalocyanine derivatives.

Compound	Forward step / mA		Reverse step / mA	
	Slop	Intercept	Slop	Intercept
**1**	-0.531	-0.0631	1.020	0.00596
**2**	-0.358	-0.106	1.100	-0.00274
**3**	-0.525	-0.0360	0.670	-0.000172
**4**	-0.322	-0.0632	0.698	0.00547

Chronocoulometry was measured by a chronoamperometry treatment in which the current response was integrated to give a response of the charge. The charge-time curve of the forward chronocoulometry step for c is the integral of Equation (1):

(2) $$Q=\frac{2{\mathrm{nFACD}}^{1/2}t^{1/2}}{\pi^{1/2}}=2{\mathrm{Kt}}^{1/2}$$

This is called the Anson Equation (2).

The reverse step is the following equation:

(3) $$Q_{r}=\frac{2{\mathrm{nFACD}}^{1/2}}{\pi^{1/2}}\{\tau^{\;1/2}+{\left(t-r\right)}^{1/2}-t^{1/2}\}$$

**Figure 9:**
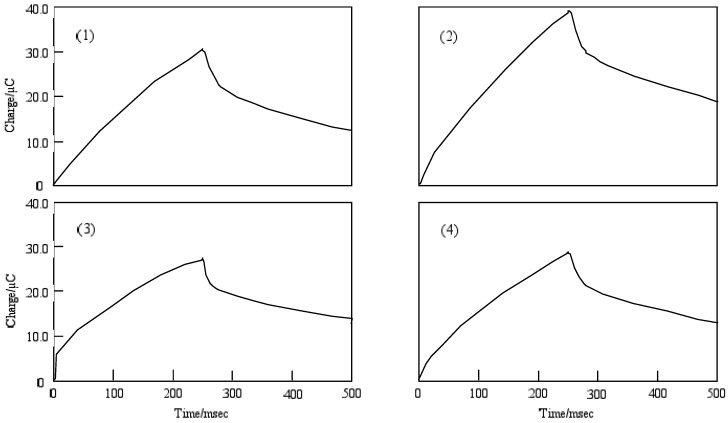
Chronocoulometry of cobalt phthalocyanine tetrasulfonic acid. Potential step; −1.2–1.6 V vs. Ag/AgCl, time interval: 250 ms. (1) Cobalt phthalocyanine tetrasulfonic acid. (2) Cobalt phthalocyanine octacarboxylic acid. (3) Cobalt octakis(hexoxy-methyl)phthalocyanine. (4) Cobalt antraquinocyanine.

Figure 9 shows the chronocoulometry of cobalt-**1**, **2**, **3** and **4**. The initial potential in each case was -1,200 mV vs. Ag/AgCl and the step width was 250 ms. The step potential was +1,600 mV vs. Ag/AgCl.

For cobalt-**1**, **2**, **3** and **4**, the extent of diffusion control increases systematically as the standard potential becomes positive. The electron charge was reached at about 30 μC in the forward step and was decreased to 15 μC, except for cobalt-**2**. In the chronocoulometry of the reduction side from -1,200 to 0 mV vs. Ag/AgCl potential, reverse steps were attenuated to 0 μC with 70 ms, except for cobalt-**2**. In the oxidation side from the 0 to +1600 mV vs. Ag/AgCl step, chronocoulometry had a linear forward step and a flat reverse step indicating no faradic activity for all compounds.

**Figure 10:**
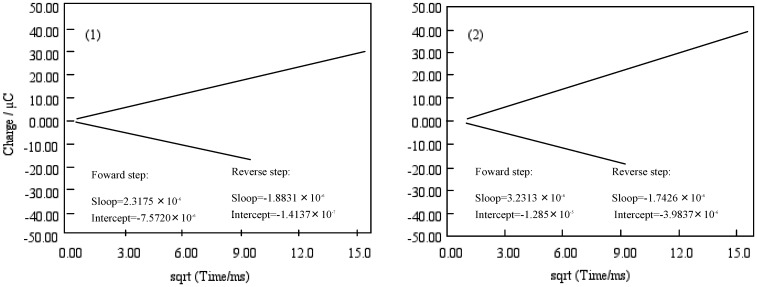
Anson plots of cobalt phthalocyanine tetrasulfonic acid (1) and cobalt phthalocyanine octacarboxylic acid (2).

Figure 10 shows Anson plots, which are converted from the charge-time curve of chronocoulometry into the relation between charge and square root of time. Only the value of solution species (Q) in three terms depends upon the scanning time. The intercept of the Anson plot expresses the sum of a double layer charging (Q_dl_) and electrolysis of adsorbed (Q_ads_). Since double step chronocoulometry is used in this work, Q_ads_ can take away Q_dl_ which is a value of the difference of intercepts between forward and reverse steps. When no adsorption of reactant or product, the intercept of Anson plot for both forward and reverse steps are Q_dl_. While reactant adsorbs but product does not, the intercept of the reverse is a measure of Q_dl_ in the presence of adsorbed reactant, and the intercept of the forward step contains both Q_dl_ and Q_ads_ for adsorbed reactant.

The chronocoulometry of cobalt-**1** and **2** shows that the reactant is adsorbed but not the product. The Q_ads_ was found from the calculation to be 7.40 and 8.92 μC for cobalt-**1** and **2**, respectively. Hence, the Q_dl_ was estimated to be 0.53 μC at 0 ms of the chronocoulometry for cobalt-**1** and **2**.

The relation between Q_r_/Q_f_ and the square root of time can be estimated by the mechanism and rate of the following chemical reaction. The value Q_r_/Q_f_ indicates the base line for the chronocoulometry of the reverse step charge Q_r_ divided by the final value of forward step Q_f_. It was found that the following chemical reaction obeyed first-order kinetics, which found the calculation to be 0.20 and 0.26 s^-1^ for cobalt-**1** and **2**. It is thought that reduction and oxidation take place in different pathways, where especially cobalt-**2** exhibits characteristic behavior.

Chronocoulometry gives rise to Q_dl_, and Q_ads_ and Q in the initial potential step:

(4) $$Q_{\;\mathrm{total}}=\frac{2{\mathrm{nFACD}}^{1/2}t^{1/2}}{\pi^{1/2}}+Q_{\mathrm{dl}}+Q_{\mathrm{abs}}$$

Q_abs_ = n F A Γ (5)

where, Q_dl_: double layer charge (C), Q_ads_: absorbed species charge (C), Γ: amount of adsorbed (mol/cm^3^).

With regards to the adsorption using equations (2) and (3), the Q_abs_ was found from calculation to be 7.40, 8.92, 2.81 and 7.07 μC for cobalt-**1**, **2**, **3** and **4**, respectively. Hence, the Q_dl_ was estimated to be 0.53 μC at 0 ms of the chronocoulometry for cobalt-**1**, **2**, **3** and **4**.

**Figure 11:**
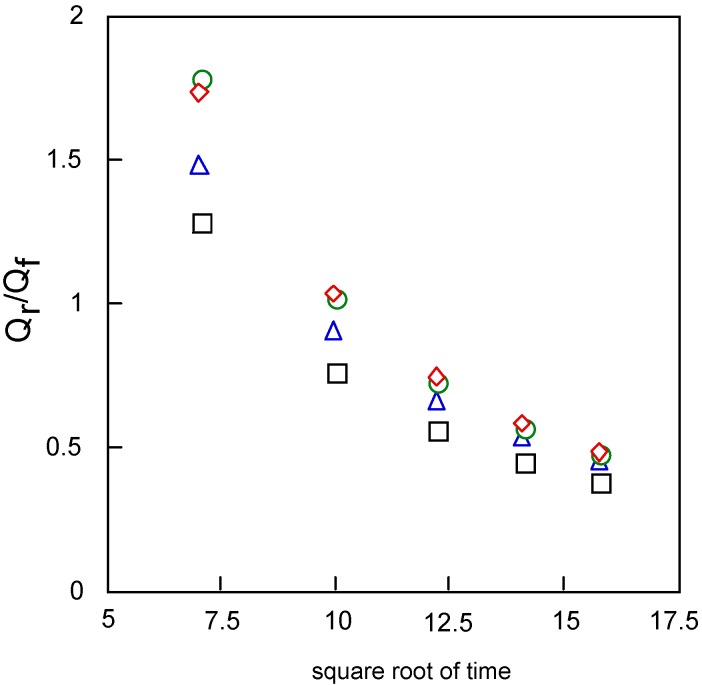
Variatuon of Q_r_/Q_f_ with squareroot of time for covalt phthalocyanine derivatives. Potential step; -1.2 – 1.6 V vs. Ag/AgCl, step width: 250 ms. Q_f_ forward step of chronocoulometry; Q_r_ forward step of chronocoulometry. □ Cobalt phthalocyanine tetrasulfonic acid; ◇ Cobalt phthalocyanine octacarboxylic acid; ○ Cobalt octakis(hexoxymethyl)phthalocyanine; △ Cobalt anthraquinocyanine.

Figure 11 shows the relationships between Q_r_/Q_f_, the baseline for the measurement of the reverse step charge Q_r_ divided by the final value of the forward step Q_f_, and the square root of time. This relationship can be estimated by the mechanism and rate of the following chemical reaction. It is found that the following chemical reaction obeyed first-order kinetics. The kinetic constants are found from calculation to be 0.20, 0.26, 0.30 and 0.30 s^-1^ for cobalt-**1**, **2**, **3** and **4**, respectively.

The oxidation of M-PCs having transition metal are electrochemically irreversible [41,46,47] and electrons are added to the orbital of phthalocyanine ring or the central metal depending on the redox potential for reduction process [47,48,49]. Consequently, it is considered that electron transfer mechanisms are proposed as the following, for cobalt-**1** [50]:

Oxidation step is:
  Co(II)-PC(SO_3_H)_4_ → [Co(III)-PC(SO_3_H)_4_]^+^ + e (6)
Reduction steps are:
  Co(II)-PC(SO_3_H)_4_+e ⇄ [Co(II)-PC(SO_3_H)_4_]^-^ (7)
  [Co(II)-PC(SO_3_H)_4_]^-^ + e ⇄ [Co(II)-PC(SO_3_H)_4_ (8)
  [Co(II)-PC(SO_3_H)_4_]^2-^ + e → [Co(I)-PC(SO_3_H)_4_]^3-^ (9)

For cobalt-**2** [50]:

Oxidation steps are:
  Co(II)-PC(COOH)_8_ → [Co(III)-PC(COOH)_8_]^+^ + e (10)
  [Co(III)-PC(COOH)_8_]^+^ → [Co(III)-PC(COOH)_8_]^2+^+e (11)
  [Co(III)-PC(COOH)_8_]^2+^ → [Co(IV)-PC(COOH)_8_]^3+^+e (12)
Reduction steps are:
  Co(II)-PC(COOH)_8_ + e → [Co(II)-PC(COOH)_8_]^-^ (13)
  [Co(II)-PC(COOH)_8_]^-^ + e ⇄ [Co(II)-PC(COOH)_8_]^2^ (14)
  [Co(II)-PC(COOH)_8_]^2-^ + e ⇄ [Co(I)-PC(COOH)_8_]^3-^ (15)

For cobalt-**3** [51,52]:

Oxidation steps are:
  Co(II)-PC(CH_2_OC_6_H_13_)_8_ → [Co(III)PC(CH_2_OC_6_H_13_)_8_]^+^ + e (16)
  [Co(II)-PC(CH_2_OC_6_H13)_8_]^+^ → [Co(III)-PC(CH_2_OC_6_H_13_)_8_]^2+^ + e (17)
Reduction steps are:
  Co(II)-PC(CH_2_OC_6_H_13_)_8_ → [Co(I)-PC(CH_2_OC_6_H_13_)_8_]^-^ (18)
  [Co(I)-PC(CH_2_OC_6_H_13_)_8_]^-^ + e ⇄ [Co(I)-PC(CH_2_O_6_H_13_)_8_]^2-^ (19)
  [Co(I)-PC(CH_2_OC_6_H_13_)_8_]^2-^ + e ⇄ [Co(I)-PC(CH_2_OC_6_H_13_)_8_]^3-^ (20)

For cobalt-**4** [51,52]:

Oxidation steps are:
  Co(II)-AQC → [Co(III)-AQC]^+^ + e (21)
  [Co(III)-AQC]^+^ → [Co(III)-AQC]^2+^ + e (22)
Reduction steps are,
  Co(II)-AQC + e ⇄ [Co(II)-AQC]^-^ (23)
  [Co(II)-AQC]^-^ + e ⇄ [Co(I)-AQC]^2-^ (24)

**Figure 12:**
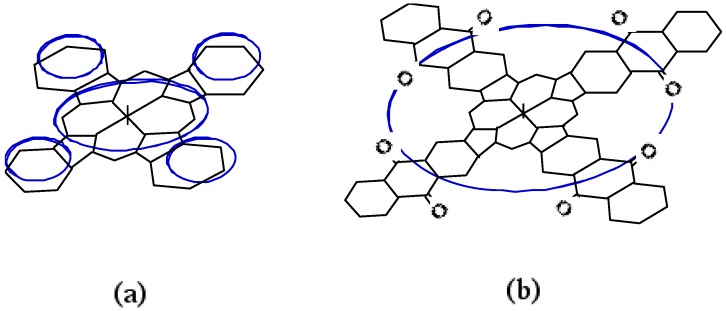
Ring current model of phthalocyanine ring and anthraquinocyanone ring. (a) Five-loop model. (b) Single-loop model.

The chemical reaction following the reversible redox process is an interaction between the anion of the phthalocyanine ring and a solvent molecule. It is thought that the ring current of M-PCs such as **1**, **2** and **3** consists of five components, corresponding to one porphyrazine and four phenylene rings, as shown in Figure 12(a).

The molecules of **4** are also planar, symmetrical and possess a highly aromatic organic system due to the presence of its highly conjugated π electron system. For this reason, **4** can be expected to have an effect on the electron transfer for anthraquinone units in the molecules. Anthraquinone-like properties were not observed for **4**, which showed reversible two step one-electron redox reactions, and were similar to **1** and **2**, so the anthraquinone units in **4** apparently had no effect on the electron transfer ability in itself, so we may conclude that the ring current constituents of **4** are different from those of M-PCs. It is believed that the π electron ring current of metal anthraquinocyanines is not made up of five loops. As the **4** are also conjugated planer molecules, the π electrons of the anthraquinone units are incorporated into the macrocyclic conjugated path of the porphyrazine ring shown in Figure 12(b). As a result of the formation of a simple loop current on **4**, the redox property of anthraquinone covers the macrocyclic delocalized π electrons.

The important parameters of a CV are the reduction and oxidation potentials for irreversible peaks, and the mid-point potential for a reversible couple, E_mid_ (Table 7). Before separation of positional isomers, the reduction and oxidation potentials of **5c** are sorted into six irreversible peaks.

After separation, fractions 1 - 3 have one pair of reversible oxidation potential and four irreversible peaks. Fraction 4 has one pair of reversible oxidation and three irreversible reduction waves. The reduction and oxidation of M-PCs are due to the interaction between the phthalocyanine ring and the central metal [49,53,54].

**Table 7:** Redox potentials of **5c** and its regioisomers.

Compound		Potential / V vs. Ag/AgCl					
		Reduction				Oxidation	
**5c**, before regioisomer separation		-0.97*	-0.71*	-0.45*	0.15*	0.37*	0.93*
Fraction 1		-1.00*	-0.58*	-0.24*		0.44	0.93*
	ΔE**					0.17	
Fraction 2		-1.05*	-0.60*	-0.19*		0.37	0.90
	ΔE**					0.10	
Fraction 3		-0.96*	-0.65*	-0.22*		0.37	0.89*
	ΔE**					0.13	
Fraction 4		-0.87*	-0.63*	-0.21*		0.34	
	ΔE**					0.01	

Potentials of reversible wave are midpoint of anodic and cathodic praks for each couple, E_1/2_.

*Irreversible peak. ** The anodic peak to cathodic peak separation for reversible couple.

The porphyrazine ring in the molecules of metal phthalocyanine derivatives or analogues is influenced by the π electrons around the closed system [16,27,32,51,55]. Although the π electron systems of **5c** and fractions 1-4 consist of one porphyrazine, two pyridinoids and two didecyl- substituted phenylene rings, the locations of these rings, except for the porphyrazine, are different for each positional isomer. The irreversible peaks are attributed to the oxidation of the central metal and the reversible couples represent the redox of the phthalocyanine ring [53].

Substituents and pyridinoid rings influenced the π electron environment in the compounds **5c** and fractions 1-4. It is thought that the effect of pyridinoid rings gives rise to changes of the electron density of the M-PCs. The difference of reduction and oxidation peaks between fractions 1-4 is attributed to the effect of the variation of the interaction between the central metal and the alkylbenzoporphyrazine. Then, the difference of CV between the **5c** and fractions 1-4 is also the effect of the interaction, since **5c** is a mixture of its positional isomers.

The ΔΕ values are the anodic peak to cathodic peak separation located in the oxidation potential region. The ΔΕ values are around 100 mV and the redox processes are the same for positional isomers, except for fraction 4. This means that the electron process of position isomers between fractions 1-3 involve approximately one electron transfer. The ΔΕ values of fraction 4 show different behavior in comparison to the others. It is thought that the different behavior for fraction 4 is attributable to the mixture of two types of *C_2v_* positional isomers. In other words, the reduction and oxidation potentials of fraction 4 are based on the interaction between two types of *C_2v_* positional isomers. No observation on the reversible couple in **5c** resulted in any interaction between the positional isomers.

**Figure 13:**
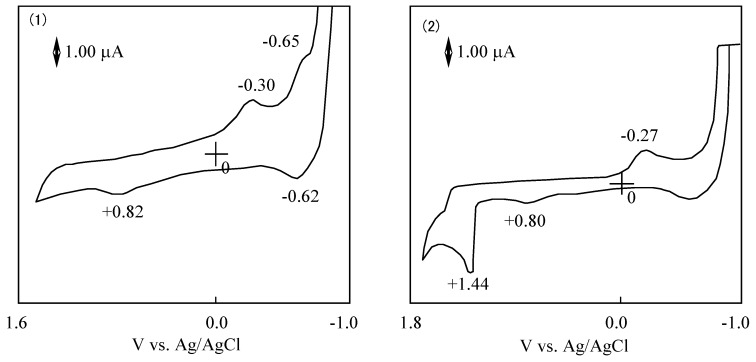
Cyclic voltammograms of subphthalocyanine derivatives in acetonitrile with tetrabutylammonium perchlorate. (1) Subphthalocyanine **6a**. (2) hexakis(*S*-phenyl)hexa-fluorosubphthalocyanine **6d**.

The potential difference between the reduction and oxidation is expressed in the HOMO - LUMO energy gap [41]. The values of λ_max_ in the Q band correlated with the potential difference between the reduction and oxidation. Figure 13 shows CVs of **6a** and **6f**. The reduction and oxidation potentials of **6** are summarized in Table 8. The reported potentials are the midpoint potential of the anodic and cathodic peaks for each couple E_mid_, and the peak potential for an irreversible step.

**Table 8:** Reduction and reduction potentials of **6**.

Compound		Potential / Vvs. Ag/AgCl				
		Reduction		Oxidation		
**6a**		-0.64	-0.30*		0.82*	
	ΔE**	0.03				
**6b**		-0.51*		0.25*	0.79*	
**6c**		-0.56*	-0.28*		0.83	
	ΔE**				0.03	
**6d**		-0.61*	-0.37*	0.18*		
**6e**			-0.27*		0.80*	1.44*
**6f**		-0.57*	-0.36*	0.18*		

Potentials of reversible wave are midpoint of anodic and cathodic praks for each couple, E_1/2_. *Irreversible peak. ** The anodic peak to cathodic peak separation for reversible couple.

The cyclic voltammogram of **6a** showed two cathodic peaks at −0.30 and −0.65 V vs. Ag/AgCl, and two anodic peaks at 0.82 and −0.62 V vs. Ag/AgCl. Compound **6a** has two irreversible oxidation and reduction at 0.82 and −0.30 V vs. Ag/AgCl, and one pair of reversible reduction potential at −0.64 V vs. Ag/AgCl.

The reduction and oxidation behavior of M-PCs are due to the interaction between the phthalocyanine ring and the central metal [55]. In the case of M-PCs having cobalt as the central metal, phthalocyanine ring oxidation occurred after the central metal, and this reduction was followed by the reduction of the central metal [50,54,56].

In the case of M-PCs, the porphyrazine ring in the molecule is influenced by the π electrons around the closed system. The π electron system of M-PCs consists of one porphyrazine and four phynylene rings [27,32,50]. Substituents on the M-PCs influence the π electron environment in the molecule, especially the four phynylene rings. It is thought that the effect of substituents gives rise to the change of electron density of the four phynylene rings in the molecules of M-PCs. Electron transfer properties of M-PCs depend on the kind of the substituents.

In the case of **6**, redox potentials had various values, but one irreversible reduction potential certainly appeared around −0.3 V vs. Ag/AgCl. In SubPC, the reduction potentials showed at −0.64 V vs. Ag/AgCl and the oxidation potential appeared at 0.82 V vs. Ag/AgCl. It is concluded that the irreversible peaks around −0.3 V vs. Ag/AgCl can be attributed to the reduction of the subphthalo-cyanine ring. The difference of cyclic voltammogram between SubPCs is attributed to the variation of the substituents that depends on the SubPC ring.

### 2.3. Photochemistry

**Figure 14:**
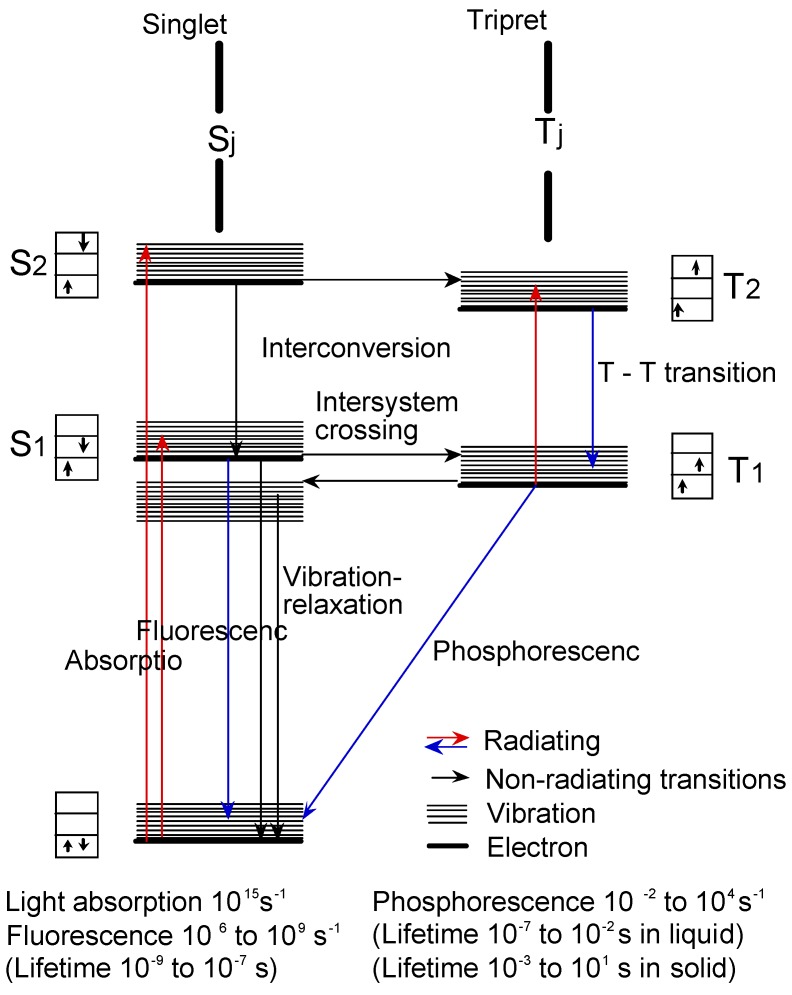
Jablonski diagram.

Figure 14 shows the Jablonski diagram, which is a scheme of radiationless transition process and emission of radiation (fluorescence and phosphorescence). In general, photochemical reactions occur in triplet states. Triplet state lifetimes are known to be between 100 ns and 10 s. The lifetime of excited singlet states is too short for a typical chemical reactions. Photochemical properties were measured the triplet state lifetime using laser-flash photolysis.

**Figure 15:**
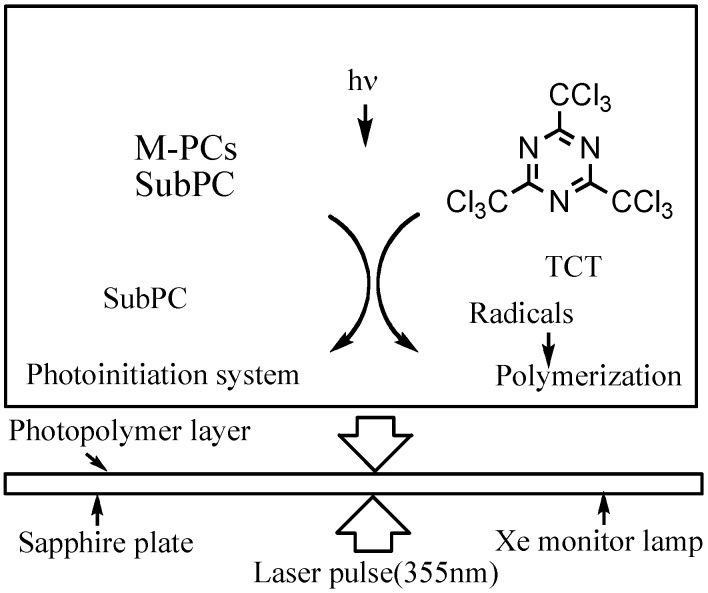
Laser flash photolysis in film.

Laser-flash photolysis in film was performed using a total reflection sapphire cell (10 · 30 mm, 1 mm thickness, and both side were cut at a 45 degree angle) [57,58], which was spin-coated with a thick of 1.2 μm SubPC containing 10% poly(methylmethacrylate) (PMMA) in cyclohexane photopolymer film as shown in Figure 15. The excitation light pulse from a Spectron Laser System Model SL402 YAG laser was expanded five-fold over the entire sample cell. A xenon lamp was used as a monitoring beam [57,58].

The measurement was repeated five times with less than 3 wt% of **6** and 2,4,6-tris(trichloromethyl)-1,3,5-triazine (TCT) as a quencher; more than 90% of the 355 nm-laser light absorbed in the sample film was absorbed by **6**.

Films were prepared as follows; the compounds **6** were added to this solution either by dissolving the dyes directly in polymer solution or by mixing a potion of concentrated dye solution. Films were adjusted to be 1.2 μm thick by spin-coating a solution onto a sample cell.

The photosensitivity of the photoinitiator system was measured as follows; the sensitive layer which was prepared by coating a cyclohexane solution of the photosensitive composition containing 5 wt% of the **6**, 5 wt% of TCT, 45 wt% of trimethylrolpropane triacrylate and 45 wt% of PMMA (90:10 mol%, MW = 50,000) onto a grained aluminium plate and exposed at 550 nm by the use of a xenon lamp, which was isolated using a monochrometer.

**Figure 16:**
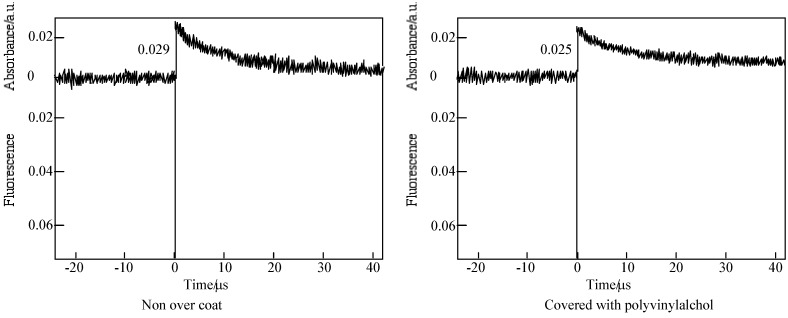
Decay trace of **5c** in PMMA film on 560 nm. Excitation wavelength: 355 nm in the presence and absence of PVA over coatings.

In the case of laser-flash photolysis of **5c** [59], the films were prepared as follows: a 10 wt% PMMA solution was made up in cyclohexanone, allylbenzopyridoporphyrazines were added to this solution by dissolving to a thickness of 1.2 μm thick by spin-coating a solution onto a sapphire cell. After that the films were covered with a poly(vinyl alcohol) (PVA) solution. Figure 16 shows the time profiles of the triplet state for one of the alkylbenzopyridoporphyrazines, **5c** in PMMA was observed using laser-flash photolysis.

The triplet state lifetimes of alkylbenzopyridoporphyarazines, **5b** - **5d** and **5e** are summarized in Table 9.

**Table 9:** Triplet lifetime of alkylbenzopyridoporphyarazines, **5b** - **5d** and **5e**.

Compound	Q band / nm	Lifetime / μs	
	in PMMA film	Non over coat	Over coat
**5b**	675.2	11.4	51.8
**5c**	717.6	10.1	46.9
**5d**	670.0	5.7	18.2
**5e**	703.9	2.6	17.9

In each alkylbenzopyridoporphyrazine, it is shown that **5b** and **5c** have longer triplet lifetimes than **5d** and **5e**. The length of the triplet lifetime for alkylbenzopyridoporphyrazine depends upon its molecular structure. The triplet lifetime of alkylbenzopyridoporphyrazines increased with increasing pyridine numbers in the molecule. It seems that if tetrapyridoporphyrazine **5a** can be soluble in common solvents and measured for laser-flash photolysis, its triplet lifetime will be shown the longest value.

The photoexcited triplet state lifetimes of **5b** and **5c** in PMMA without a PVA coating were estimation to be 11.4 and 10.1 μs, respectively. While covered with a PVA coating, the photoexcited triplet state lifetimes of **5b** and **5c** were estimated as 51.8 and 46.9 μs, respectively. Compared with each compound, the triplet state lifetime in PMMA covered with PVA was longer than without the PVA coating.

The Q band absorption of alkylbenzopyridoporphyrazines in PMMA films was similar to that in solution, but the profile of Q band in PMMA film became wider than that in solution and moved to a longer wavelength, except for **5e**.

Non-transition M-PCs were known to be excellent photosensitizers because of their chemical stability and high absorbance in the 650–700 nm region [60]. In the presence of a photosensitizer, photooxidation progresses via singlet state oxygen [61,62,63,64,65,66,67,68,69,70,71]. M-PCs in the excited triplet state react with ground triplet state dioxygen. The triplet state dioxygen generated singlet excited state oxygen. The singlet excited oxygen reacts with a substrate to produce oxide, Equations 25 - 27 [33,72].

(25) $$\mathrm{M-PC}\;\;\;\overset{h\nu }{\to }\;\;{\;}^{1}\mathrm{M-PC}\;\;\;\overset{\text{intersystem}\;\text{crossing}}{\to }\;\;{\;}^{3}\mathrm{M-PC}$$

^3^M-PC + ^3^O_2_ → M-PC + ^1^O_2_ (26)

^1^O_2_ + substrate → substrate oxide (27)

Both covered with a coating and without PVA, the photooxidation proceeded by the same mechanism. However, in the case of being covered with PVA, the photoexited triplet state lifetimes of alkylbenzopyridoporphyrazines, **5b** - **5d** and **5e**, were longer than in the case of non-overcoating with PVA. In the case of non-coated with PVA the shorter decay time was considered due to M-PC quenching by oxygen existing in an air atmosphere. While under the coated state with PVA, they suppressed the oxygen-permeation from the air atmosphere into the photopolymer layer. As a result, grand triplet state dioxigen was not furnished from the surrounding to the system. Alkylbenzo-pyridoporphyrazines in the case of being covered with PVA behaved as a model for a practical photosensitizer in tumors or cancer cells.

In comparison with **5b** and **5c**, the photoexcited triplet lifetime of **5b** was slightly longer than **5c**, the absorption intensities for **5c** were stronger than **5b**. So that in these aspects, there is little to choose as a sensitizer for PDT between the two. As Py rings in the molecule of alkylbenzo-pyridoporphyrazines increased, the water-solubility is expected to increase. In the case of the *N,N´,N´´,N´´´,N´´´´*-tetramethylated quaternized forms of tetrapyridoporphyrazines, it was reported that the complexes do not form an aggregation in an aqueous solution [72,73,74]. Although the long alkyl-chain substituents in **5b** and **5c** will occur in aggregation, **5b** and **5c** are expected to rapidly undergo photodecomposition after the photooxidation process, similar to alkyl phthalocyanine derivatives [9].

Consequently, since **5b** and **5c** have the most intense absorption and a longer triplet state lifetime, we think **5b** and **5c** could become useful sensitizers for PDT. The photosensitizer should be made in isomerically pure form [9]. Isomers of **5b** have not been reported yet, but **5c** has been separated and identified [33]. Thereupon, regioisomers of **5c** were examined by laser- flash photolysis, and **5b** will be reported in the near future. As mentioned above, the regioisomers of the compound, **5c** were separated into four green- to blue-colored fractions by TLC [33]. The four fractions have a different ^1^H-NMR, UV-VIS and fluorescent spectra. The four fractions separated by TLC have been attributed to four of the five possible region- isomers of **5c**.

**Figure 17:**
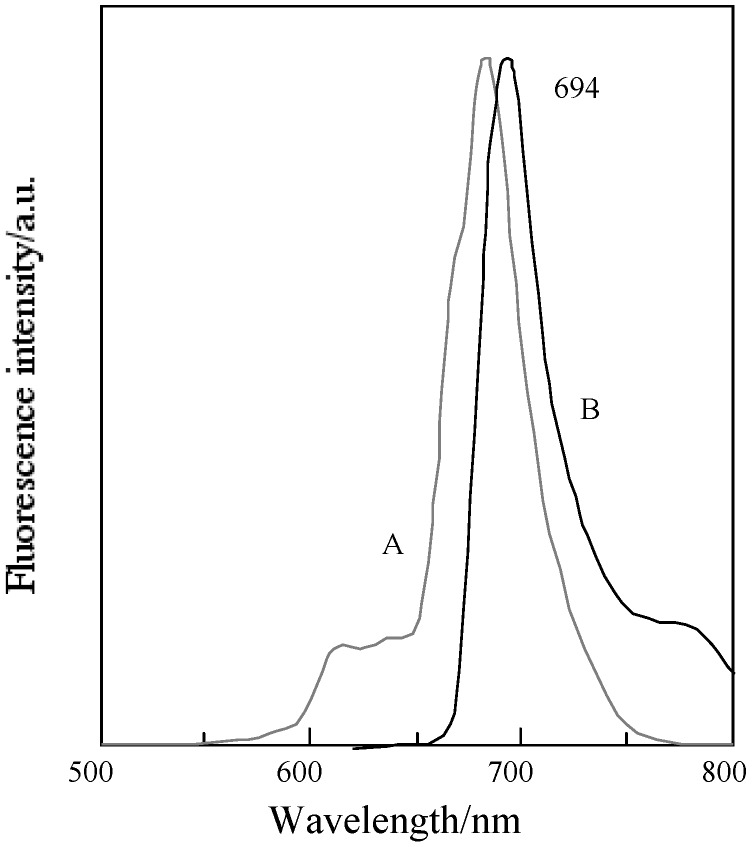
Fluorescence and excitationspectra of **5c** in DMF. A; Excitation spectrum, B; Fluorescence spectrum.

Figure 17 shows the fluorescence and excitation spectra of **5c**. The excitation spectra of **5c** and its fractions have almost the same profile. No significant change on the fluorescence spectra was observed for **5c** and its fractions.

Table 10 shows the Q band and fluorescent maximum of fractions of **5c**. The assignment of the Q band from each fraction was carried out on the theory of the relationship between symmetry and the Q band [1,4,74,75,76,77]. The Q band splits into two peaks of the highest isomer symmetry, the splitting Q band is decreased with a decreasing symmetry [78]. The symmetry of the position isomer of **5c** was decreased in orders of *C_2h_, D_2h_, C_2v_, C_s_* [79]. The regioisomers of **5c** had molecular structures with *D_2h_*, *C_2h_*, *C_s_* and *C_2v_* symmetry for fractions 1, 2, 3 and 4, respectively [33]. Two types of *C_2v_* isomers could not be isolated [33].

**Table 10:** Absorption and fluorescence maxima for each fraction of **5c** in solution.

Compound	Symmetry	Q band /nm	Fluorescence / nm
Fraction 1	*D2h*	627, 690, 705	704
Fraction 2	*C2h*	609, 673, 708	706
Fraction 3	*Cs*	619, 689	695
Fraction 4	*C2v*	610, 638, 671, 688	701

Under line; mostintense peak.

Table 11 shows the photoexcited triplet lifetime of fractions separated from **5c**. In spite of the presence or absence of PVA coatings, the triplet lifetime was increased with a decreasing symmetry of position isomers, which were ordered as *C_2h_, D_2h_, C_2v_* and *C_s_* for fractions 2, 1, 4 and 3, respectively. The photoexcited triplet state lifetimes of fraction 3 in PMMA absence and presence of a PVA coating were estimated to be 14.29 and 25.97 μs, respectively. Of each fraction except for fraction 3, the length of the lifetime was shorter than **5c**, and the sensitivities of triplet-triplet (T-T) absorptions were observed as very low.

**Table 11:** Triplet lifetime of each fraction of **5c**.

Compound	Q band / nm	Lifetime / μs	
	in PMMA film	Non over coat	Over coat
Fraction 1	782.2, 681.6, 653.5	7.0	11.3
Fraction 2	670.0	0.9	1.6
Fraction 3	696.4	14.29	25.97
Fraction 4	665.5	6.4	9.2

Although the length of the triplet lifetime of **5c** was observed to be approximately four times that seen in the absence of a PVA overcoating, the fractions of **5c** were only had about 1.5 times as long as triplet lifetimes in comparison with the absence and presence of PVA coatings.

Unfortunately, a precise explanation for this cannot be provided, but it seems to be due to the following phenomenon: phthalocyanine derivatives were well known to aggregate in water and non-coordinating solvents. Zinc non-peripheral phthalocyanine derivatives having long side chains formed an aggregation at least 10^−5^ mol·L^-1^ in cyclohexane [9,78]. It is enough thought that the samples in this study for laser-flash photolysis were formed in aggregation in the experimental condition. The aggregation degrees for **5c** and its isomers are different from each other, so the aggregation ability of **5c** and each of its isomers is different and complicated. Since compounds **5b** and **5c** consisted of mixtures of their isomers, the aggregation and relationships of the energy levels between samples and the triplet state of dioxygen became a complication. For this reason, compounds **5b** and **5c** could give relatively long lifetimes. The molecular structure of fraction 3 is suitable to occur in the T-T absorption in the system.

In order to estimate the photoexcitation mechanism, the triplet lifetime of each fraction was measured, containing *N,N’*-tetramethyl-4,4’-diaminobenzophenone (Michler’s ketone) as an additional quencher. Using Micher’s ketone, the lifetimes without PVA coatings were estimated as 21.19 and 14.03 μs for fractions 3 and 4, respectively (Table 12). These values of lifetime were longer than in the absence of Micher’s ketone. In the case of fractions 1 and 2, no T-T absorption occurred. The results were thought to be that each fraction has different energy levels of grand and excited states. The T-T absorption took place via the interactions between the energy levels of grand or excited states of fractions and the triplet of dioxygen or Michler’s ketone.

**Figure 18:**
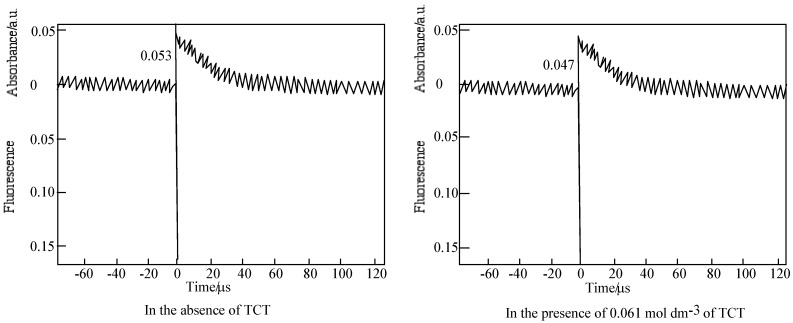
Decay trace of **6** in PMMA film on 450 nm. Excitation wavelength: 355 nm in the presence and absence of TCT. Substance concentration; 0.15 mol·L^-1^; Excitation wavelength; 355 nm.

The time profiles of the triplet state for **6** in PMMA film are shown in Figure 18. The fluorescence peak and transient decay were observed upon the excitation at 355 nm pulse of a PMMA film containing **6**. The photoexcited triplet state lifetime of **6** was estimated for 20 s laser pulse. The transient absorption was assigned to a triplet state [78].

**Table 12:** Triplet lifetime of each fraction of **5c** using Micher’s ketone as a quencher.

Compound	Lifetime / μs	
	Non over coat	Over coat
Fraction 1	-	-
Fraction 2	-	-
Fraction 3	21.19	72.72
Fraction 4	14.03	47.32

**Figure 19:**
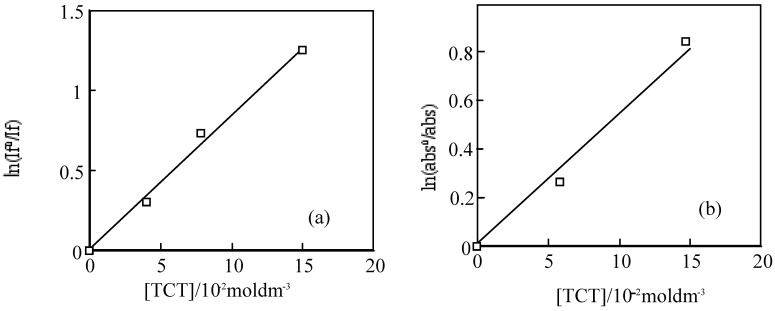
Quenching of **6** by TCT in PMMA. (a) Fluorescence on 572 nm. Substance concentration: 0.03 mol·L^-1^; Excitation wavelength: 550 nm; (b) Triplet absorption intensity on 450 nm. Substance concentration: 0.15 mol dm^−3^; Excitation wavelength: 355 nm.

For the laser excitation of PMMA film at excitation of 550 nm using fluorescence spectrometer, the strongest fluorescence of **6** was efficiently quenched by TCT The fluorescence intensity was reduced with the concentration of TCT from 0 to 0.15 M. The fluorescence intensity is in direct proportion to the concentration of the TCT [80], the relationship gives a good straight line and it exhibits the Perrin type static quenching process [81] (Figure 19). The quenching radius (R_f)_ was determined from the slope of the logarithmic plot, and was estimated to be 15 nm.

The sensitization mechanisums between SubPC derivatives and TCT are thought as equations (28–41), where I_f_^0^ and I_f_ are the fluorescence intensities in the absence and presence of the quencher TCT, τ_f_^0^ is the lifetime in the absence of quencher, k_q_ is the dynamic quenching rate constant, k_q_^i^ is the static quenching constant, V is the quenching shere, V = 4πRf/3, N is Avogadro's number, Rf is the quenching radius, X = K_i_Σ[(SubPC−(i−1)TCT)] + 1. The mutistep equibrium between **6** derivatives and TCT is shown in Equations (28) and (29):

(28) $$[\mathrm{Subpc}-(i-1)\mathrm{TCT}\;\;\;+\;\;\;\mathrm{TCT}\overset{K_{i}}{⇄}(\mathrm{SubPC}-\mathrm{iTCT})\;\;i=1,2,3\ldots$$

(29) $$\begin{array}{ll} \mathrm{Ki}=\left[\mathrm{SubPC}-\mathrm{iTCT}\right]/\left\{\left[\mathrm{SubPC}][\mathrm{SubPC}-(i-1)\mathrm{TCT}\right]\right\} & \\ & i=1,2,3\ldots \end{array}$$

Equation (30) implies that SubPC derivatives and (SubPC–iTCT) are excited to an excited state *SubPC by absorbing laser pulse. Equations (31) and (32) mean that the *SubPC undergoes nonradiative and radiative deactivation. Then, the *SubPC reacted with TCT forms radicals such as chloro or dichloromethyl radicals as shown in Equation (33). Further, the SubPC becomes triplet state by intersystem crossing in Equation (34). The (SubPC–iTCT) also undergones (*SubPC–iTCT) by absorbing laser pulse (Equation 35). The activate state (*SubPC–iTCT) takes statically sensitized decomposition of TCT (Equation 36). Then, (*SubPC–iTCT) undergoes radiative, monoradiative deactivation and intercrossing as described in Equatons (38) and (39). The attention of fluorescence with the concentration of TCT in film is described as Equation (40). When (*SubPC–iTCT) does not emit fluorescence, meaning k_q_^i^ is much larger than any decay rate. Equation (40) is described Equation (41) of the dynamic and static quenching terms.

(30) $$\mathrm{SubPC}\;\;\;\overset{h\upsilon }{\to }\;\;{\;}^{*}\mathrm{SubPC}$$

(31) $${\;}^{*}\mathrm{SubPC}\;\;\;\overset{k_{d}}{\to }\;\;\;{\;}^{*}\mathrm{SubPC}$$

(32) $${\;}^{*}\mathrm{SubPC}\;\;\;\to \;\;\;\mathrm{SubPC}+{\;\;\;}^{{h\upsilon }_{f}}$$

(33) $${\;}^{*}\mathrm{SubPC}\;\;\;+\;\;\;\mathrm{TCT}\;\;\;\overset{k_{q}}{\to }\;\;\;\mathrm{SubPC}+\;\;\;\mathrm{radicals}$$

(34) $${\;}^{*}\mathrm{SubPC}\;\;\;\to \;\;\;\mathrm{Triplet}$$

(35) $$\left(\mathrm{SubPC}-\mathrm{iTCT})\;\;\;\overset{h\upsilon }{\to }\;\;\;({\;}^{*}\mathrm{SubPC}-\mathrm{iTCT}\right)$$

(36) $$({\;}^{*}\mathrm{SubPC}-\mathrm{iTCT})\;\;\;\to \;\;\;(\mathrm{SubPC}+(i-1)\mathrm{TCT})\;\;+\;\;\mathrm{radicals}$$

(37) $$({\;}^{*}\mathrm{SubPC}-\mathrm{iTCT})\;\;\;\to \;\;\;(\mathrm{SubPC}-\mathrm{iTCT})\;\;+\;\;{h\upsilon }_{f}$$

(38) $$\left({\;}^{*}\mathrm{SubPC}-\mathrm{iTCT})\;\;\;\overset{k_{d^{i}}}{\to }\;\;\;(\mathrm{SubPC}-\mathrm{iTCT}\right)$$

(39) $$\left({\;}^{*}\mathrm{SubPC}-\mathrm{iTCT})\;\;\;\overset{k_{{\mathrm{isc}}^{i}}}{\to }\;\;\;(\mathrm{Triplet}-\mathrm{iTCT}\right)$$

I_f_^0^ = {(1 + k_q_ τ f^0^[TCT])(X + k_q_τf^0^)} / {X + k_q_τf^0^[TCT] + [Xk_q_^i^τf^0^ − k_q_τf^0^[TCT]exp(-VN[TCT])} (40)

I_f_^0^/I_f_ = (1 + k_q_ τ f^0^[TCT]exp(VN)[TCT]) (41)

The triplet state of **6** in a PMMA films was also quenched by TCT. The initial absorptions were decreased with the concentration of TCT. The triplet life times measured almost the same. The logarithmic plots of the initial absorption at various concentration of TCT were fitted to the Perrin equation, the triplet quenching radius (Rt) were calculation to be 1.3 nm, which was almost same as R_f_. The results indicate the triplet initial absorption was not quenched by TCT, since the triplet state generally came from the fluorescent singlet state, and the triplet is apparently reduced with the decrease of the singlet state.

The reduction and oxidation behavior of M-PCs are due to the interaction between the phthalocyanine ring and the central metal [40]. In the case of M-PCs, the porphyrazine ring in the molecule is influenced by the π electrons around the closed system. The 18−π electron system of M-PCs consists of one porphyrazine and four phynylene rings [27,32,50]. Substituents of M-PCs influence the 18 π electron environment in the molecule of the four phynylene rings, and are dependent on electron transfer properties of M-PCs. In the case of SubPC and its derivatives, redox potentials had various values. This implies the possibility of an electron transfer from the singlet to an excited state of **6** to the higher TCT state. The derivatives of **6** derivatives present similar phenomena to **6**. It is suggested that the static-singlet-quenching process from the singlet photoexcited **6** to TCT is predominant in the sensitization.

### 2.4. Near infrared absorptions of ***15***

**Figure 20:**
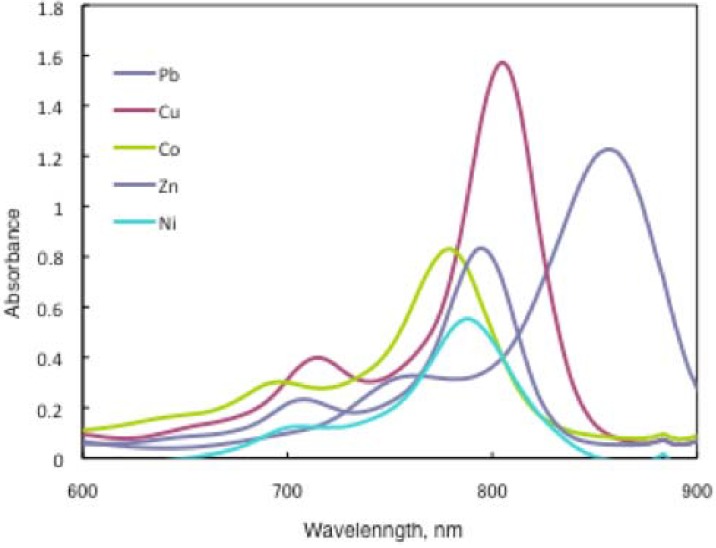
UV-Vis spectra of **15a**.

**Figure 21:**
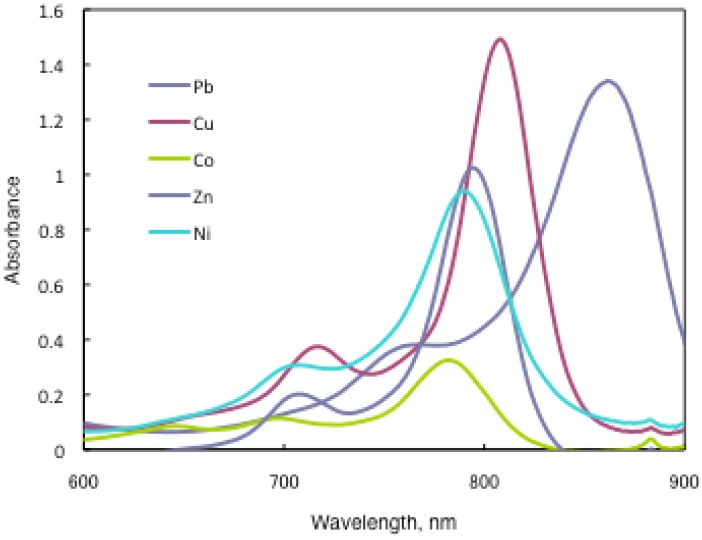
UV-Vis spectra of **15b**.

**Figure 22:**
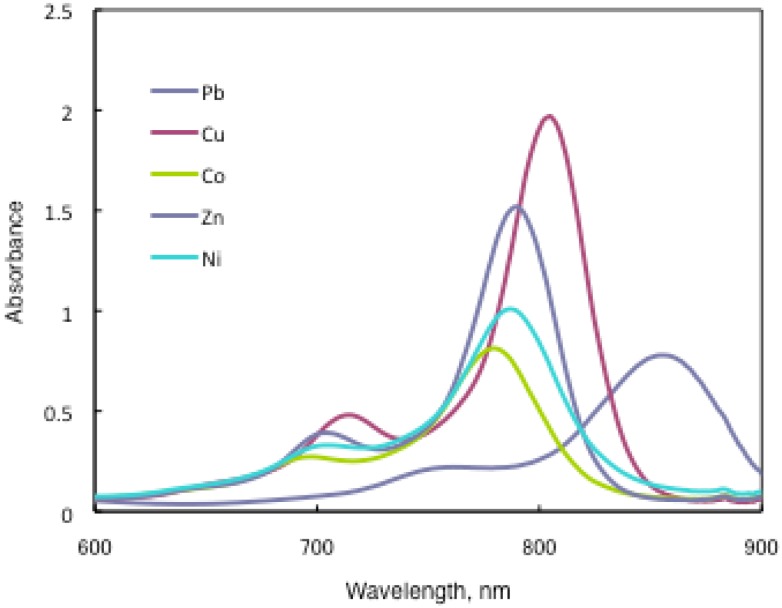
UV-Vis spectra of **15c**.

In substituted M- and H_2_-PCs, a strong absorption, termed the Q band, is detected in the visible region between 650 and 690 nm, and another in the UV-Vis between 320 and 370 nm, called the Soret band. A typical value for the extinction coefficient (ε) of the Q band is around 10^5^ cm^2^· mol^−1^. The absorption spectra of the synthesized compounds **7** show typical shapes for M-PCs (Figure 20, Figure 21 and Figure 22). They also displayed strong absorption peaks in the visible region at around 800 nm. The strongest peaks in the visible region are assigned as the Q band, which were attributed to the allowed π−π^∗^ transition of the phthalocyanine ring. The Q band absorption of synthesized compounds **15** shifted by 100–150 nm to a longer wavelength in comparison to unsubstituted M-PCs.

In the case of **15a**, absorption maxima are moved to longer wavelengths in the order of Co, Ni, Cu, Zn, and Pb. Compounds **15b** and **15c** show similar phenomena. In comparison to the same central metal, non-peripheral substituted phthalocyanines, **15a**, **15b**, and **15c** show longer wavelengths than peripheral compounds. Metal-free **15a**, **15b**, and **15c** were also synthesized. The Q band absorption peaks of **15a**, **15b**, and **15c** respectively appeared at longer wavelengths of 815, 820, and 816 nm. In general, H_2_-PCs show shorter wavelengths than corresponding M-PCs. Metal-free **15a**, **15b**, and **15c**, which have remarkably bulky substituents, increase the distortion of the molecule because the four central cavity sizes cannot be fixed. Then, the Q band of metal-free **15a**, **15b**, and **15c** were not split. The Q band is known to split into two peaks for high symmetry; the splitting Q band decreases with decreasing symmetry. Metal-free **15a**, **15b**, and **15c** display decreased symmetry as a result of the molecular distortion. We leave a detailed discussion about these phenomena of Q band of metal-free **15a**, **15b** and **15c** for another opportunity. The Q band absorption data of **15** are presented in Table 13.

**Table 13:** The Q band absorption data of **15**.

Solvent	Central metal	15a	λmax / nm log ε 15b	15c
Chloroform	Metal-free	815 5.22	820 5.33	816 5.08
Chloroform	Pb	857 5.09	862 5.13	855 4.89
Chloroform	Cu	805 5.20	808 5.17	805 5.29
Chloroform	Ni	788 4.74	790 4.97	787 5.00
Chloroform	Zn	795 4.92	795 5.01	789 5.18
Chloroform	Co	779 4.91	783 4.51	780 4.91
Toluene	Pb	838 5.15	846 5.18	836 5.08
Toluene	Cu	791 5.01	796 5.06	790 5.01
Toluene	Ni	777 5.09	782 5.10	776 4.94
Toluene	Zn	792 4.20	790 4.34	782 4.86
Toluene	Co	775 4.71	778 4.89	774 4.89

The Q band shifts depend upon the change in the electron distribution in the phthalocyanine ring caused by substituents and their position. These results suggest that the steric hindrance arising from the substituted *S*-aryl groups appears to be as significant, as reported by Kobayashi [39]. However, a difference of the Q band in **15** is low between substituents, methyl, methoxy and *tert*-butyl. In this case, electron-donating substituents only slightly affect the movement to a longer wavelength. Although the effect of the central metal on the energy of Q band is usually small [20], absorption maxima of **3** are moved to longer wavelengths, and apparently increase with the ionic radius of the central metal, particularly lead. Lead complexes of **15** showed amplified structural distortion.

Because non-peripheral *S*-aryl substituted phthalocyanines having Pb as the central metal have an absorption band near 500 nm, **15a-Pb**, **15b-Pb**, and **15c-Pb** give red solutions; the other central metals of **15a**, **15b**, and **15c** give slightly reddish solutions.

## 3. Experimental Section

### 3.1. Equipment

UV-Vis spectra were measured on a Shimadzu UV-2400PC spectrometer. Each sample was prepared in toluene at 5.0 × 10^−5^ mol·L^-1^, in chloroform at 5.0 × 10^−5^ mol·L^-1^, in Py at 5.0 × 10^−5^ mol ·L^-1^. Fluorescent spectra were recorded in DMF on a Nihon Bunko Jasco FP-6600 spectrofluorometer. The ^1^H-NMR spectra were measured at 400 MHz on a Bruker Avance 400S and 90 MHz on a Nihon Denshi Joel EX-90 in dimethylsulfoxide-*d*_6_ (DMSO-*d*_6_), benzene-*d*_6_ or chloroform-*d* (CDCl_3_) using tetramethylsilane (TMS) as the internal standard. Elemental analysis was carried out using a Perkin-Elmer 2400CHN instrument. Mass spectra were taken with a Nihon Denshi Joel JMS-AX500 mass spectrometer. Melting points were measured with a Stanford Research Systems MPA100 Optimelt automated system.

### 3.2. Materials

All chemicals were purchased from Aldrich or Tokyo Chemical Industry Co. Ltd. They were used as received without further purification. For chromatographic separation, silica gel was used (60, particle size 0.063–0.200 nm, 7734-grade; Merck).

### 3.3. Synthesis of phthalocyanine tetrasulfonic acids ***1-M*** (M = Fe, Co, Cu, Zn)

A mixture of 4-sulfophthalic acid (12.3 g, 50 mmol), urea (30.0 g, 0.50 mol), metal chloride (30 mmol) and DBU (1 g) as a catalyst dissolved in 150 mL of 1,2,4-trichlorobenzene as solvent was heated to 210 ºC for 180 min (Scheme 1). The product was filtered, washed with benzene until all 1,2,4-trichlorobenzene was removed and then dried under vacuum to a constant weight; **1-Fe** (75%) Found: C, 43.20%; H, 1.99%; N, 12.45%. Calcd. for C_32_H_16_N_8_O_12_S_4_Fe: C, 43.25%; H, 1.81%; N, 12.60%. IR (KBr): ν_max_/cm^−1^ 3000 (ν_C-H_), 1700 (ν_C-C_), 1450 (δ_C-H_), 1380 (ν_S-O_), 1050 (δ_C-H_), 770 (δ_C-H_). UV-Vis: λ_max_ Py/nm: 688.5, 649.0, 354.0, 213.0; **1-Co** (75%) Found: C, 42.92%; H, 2.08%; N, 12.67%. Calcd. for C32H16N8O12S4Co: C, 43.10%; H, 1.80%; N, 12.56%. IR (KBr): ν_max_/cm^−1^ 3000 (ν_C-H_), 1700 (ν_C-C_), 1450 (δ_C-H_), 1380 (ν_S-O_), 1050 (δ_C-H_), 770 (δ_C-H_). UV-Vis: λ_max_ Py/nm: 688.5, 649.0, 354.0, 213.0; **1-Cu** (75%) Found: C, 42.86%; H, 1.67%; N, 12.45%. Calcd. for C_32_H_16_N_8_O_12_S_4_Cu: C, 42.88%; H, 1.79%; N, 12.50%. IR (KBr): ν_max_/cm^-1^ 3000 (ν_C-H_), 1700 (ν_C-C_), 1450 (δ_C-H_), 1380 (ν_S-O_), 1050 (δ_C-H_), 770 (δ_C-H_). UV-Vis: λ_max_ Py/nm: 688.5, 649.0, 354.0, 213.0; **1-Zn** (75%) Found: C, 42.67%; H, 1.80%; N, 12.53%. Calcd. for C_32_H_16_N_8_O_12_S_4_Zn: C, 42.79%; H, 1.79%; N,12.47%. IR (KBr): ν_max_/cm^-1^ 3000 (ν_C-H_), 1700 (ν_C-C_), 1450 (δ_C-H_), 1380 (ν_S-O_), 1050 (δ_C-H_), 770 (δ_C-H_). UV-Vis: λ_max_ Py/nm: 688.5, 649.0, 354.0, 213.0.

### 3.4. Synthesis of phthalocyanine octacarboxylic acids ***2-M*** (M = Fe, Co, Cu, Zn) (Scheme 2)

A mixture of pyromellitic dianhydride (2.50 g, 11.5 mmol), urea (13.0 g, 0.22 mol), metal chloride (23.5 mmol) and 0.1g of DBU was heated to 250 ºC until the reaction mixture was fused. The reaction product was washed with water, acetone and 6 M-hydrochloric acid (HCl). After drying, the solid obtained was hydrolyzed. Thirty grams of crude product, 30 g of KOH and 90 mL of water were heated for 480 min at 100 ºC. The mixture was diluted with 200 mL of water and filtered. The filtrate was acidified to pH 2 with concentrated HCl . At this point the product precipitated as a blue colored solid, which was separated from the solution by a centrifuge. The solid was washed with water three times, and dried. **2-Fe** (yield: 30%) Found: C, 52.11%; H, 1.41%; N, 12.25%. Calcd. for C_40_H_16_N_8_O_16_Fe: C, 52.19%; H, 1.75%; N, 12.17%. IR (KBr): ν_max_/cm^-1^ 3300 (ν_C-H_), 2900 (ν_C-H_), 1750 (ν_C-O_), 1720 (ν_C-O_), 1480 (ν_C-C_), 1060 (δ_C-H_), 720 (δ_C-H_). UV-Vis: λ_max_ Py/nm: 684; **2-Co** (yield: 30%) Found: C, 52.44%; H, 1.74%; N, 12.25%. Calcd. for C_40_H_16_N_8_O_16_Co: C, 52.04%; H, 1.74%; N, 12.13%. IR (KBr): ν_max_/cm^-1^ 3300 (ν_C-H_), 2900 (ν_C-H_), 1750 (ν_C-O_), 1720 (ν_C-O_), 1480 (ν_C-C_), 1060 (δ_C-H_), 720 (δ_C-H_). UV-Vis: λ_max_ Py/nm: 684; **2-Cu** (yield: 30%) Found: C, 52.03%; H, 1.86%; N, 11.97%. Calcd. for C_40_H_16_N_8_O_16_Cu: C, 51.76%; H, 1.73%; N, 12.07%. IR (KBr): ν_max_/cm^-1^ 3300 (ν_C-H_), 2900 (ν_C-H_), 1750 (ν_C-O_), 1720 (ν_C-O_), 1480 (ν_C-C_), 1060 (δ_C-H_), 720 (δ_C-H_). UV-Vis: λ_max_ Py/nm: 684; **2-Zn** (yield: 30%) Found: C, 51.60%; H, 1.66%; N, 12.16%. Calcd. for C_40_H_16_N_8_O_16_Zn: C, 51.66%; H, 1.73%; N, 12.04%. IR (KBr): ν_max_/cm^-1^ 3300(ν_C-H_), 2900(ν_C-H_), 1750 (ν_C-O_), 1720 (ν_C-O_), 1480 (ν_C-C_), 1060 (δ_C-H_), 720 (δ_C-H_). UV-Vis: λ_max_ Py/nm: 684.

### 3.5. Synthesis of octakis(hexoxymethyl)phthalocyanines ***3-M*** (M=Fe, Co, Cu, Zn)

Compounds **3** were synthesized from a metal chloride and 1,2-dicyano-4,5-bis(hexoxymethyl)-benzene (**10**), which was synthesized from *o*-xylene via 1,2-dibromo-4,5-dimethylbenzene (**7**), 1,2-dibromo-4,5-bis(bromo-methylbenzene) (**8**) and 1,2-dibromo-4,5-bis(hexoxymethyl)benzene (**9**) (Scheme 3).

#### 3.5.1. Synthesis of 1,2-dibromo-4,5-dimethylbenzene (**7**)

 Bromine (500 g, 3.12 mol) was added dropwise to a mixture of *o*-xylene (166 g, 1.56 mol), iodine (2 g) and iron (3 g), during a 420 min period while stirring at 5-10 °C. After the reaction, the reaction mixture was poured into water and filtered. The precipitate was washed with a 3%-NaOH solution and recrystallized from methylcyclohexane; yield 49%. mp 86.6°C. Found: C, 36.18%; H, 2.64%. Calc for C_8_H_8_Br_2_: C, 36.66%; H, 3.08 %. IR (KBr): ν_max_/cm^-1^ 2950 (ν_C-H_), 1601 (ν_C-C_), 1440 (ν_C-C_), 1340 (ν_C-C_), 1120 (ν_C-C_), 880 (δC-H), 640 (δC-H). ^1^H-NMR (CDCl_3_): δ, ppm 2.10 (s, 6H), 7.36 (s, 2H). MS *m/z* = 264.

#### 3.5.2. Synthesis of 1,2-dibromo-4,5-bis(bromomethylbenzene) (**8**)

A mixture of **7** (30.2 g, 0.11 mol) dissolved in 100 mL of carbon tetrachloride, 42.7 g (0.24 mol) of *N*-bromosuccinimide (NBS) and 0.08 g of 2,2’-azobis(isobutyro)nitrile was heated to 80 °C and refluxed for 480 min. After removing the carbon tetrachloride, the crude product was recrystallized from heptanes and dried under vacuum; yield 21%. mp 84.7°C, Found: C, 22.72%; H, 1.15%;. Calc for C_10_H_10_Br_2_: C, 22.78%; H, 1.43%. IR (KBr): ν_max_/cm^-1^ 3000 (ν_C-H_), 1650 (ν_C-C_), 1460 (ν_C-C_), 1200 (ν_C-C_), 950 (δC-H), 620 (δC-H). ^1^H-NMR (CDCl_3_): δ, ppm 4.43 (s, 4H), 7.53 (s, 2H); MS *m/z* = 422.

#### 3.5.3. Synthesis of 1,2-dibromo-4,5-bis(hexoxymethyl)benzene (**9**)

A piece of sodium was added to 120 g (1.18 mol) of hexanol. After the added sodium dissolved, 5.00 g (0.13 mol) of **8** was added. The temperature was gradually raised to 155 °C, and then the mixture was stirred for 90 min. The excess hexanol was removed by distillation and one hundred grams of water were added to the reaction mixture to dissolve the salt by-product. The resultant mixture was extracted with diethyl ether. The organic layer was washed with a small quantity of water and dried over calcium chloride. The diethyl ether was distilled off. The product **9** is a brown liquid; yield 88%. IR (KBr): ν_max_/cm^-1^ 2900 (ν_C-H_), 1670 (ν_C-C_), 1240 (ν_C-C_), 1050(δC-H), 630(δC-H). ^1^H-NMR (CDCl_3_): δ, ppm 0.89 (s, 6H), 1.28 (m, 16H), 3.52 (s, 4H), 4.45 (s, 4H ), 7.64 (s, 2H).

#### 3.5.4. Synthesis of 1,2-dicyano-4,5-bis(hexoxymethyl)benzene (**10**)

A mixture of 15.2 g (33.8 mmol) of 1,2-dibromo-4,5-bis(hexoxymethyl) benzene (**3**), 13.6 g (96.6 mmol) of copper cyanide and 100 cm^3^ of DMF was refluxed for 300 minutes, then cooled, and poured into 170 cm^3^ of aqueous ammonia. The mixture was stirred for 10 min, and was filtered. The bluish residue was washed with water and dried. The product was extracted with diethyl ether for 1,440 min using a Soxhlet apparatus and the solvent was evaporated; yield 66%. mp 360.0°C, Found: C, 80.58%; H, 9.37%;. N, 8.26%; Calc for C_22_H_32_N_2_O_2_: C, 81.43%; H, 9.94%; N, 8.63%. IR (KBr): ν_max_/cm^-1^ 3200 (ν_C-H_), 2100 (ν_C-N_), 1460 (ν_C-C_), 1240 (ν_C-C_), 1095(δ_C-H_), 60(δ_C-H_). ^1^H-NMR (CDCl_3_): δ, ppm 4.43 (s, 4H ), 7.53 (s, 2H). MS *m/z* = 357.

#### 3.5.5. Synthesis of **3-M**

Compounds **3-M** were synthesized as follows: a mixture of 2.00 g (5.61 mmol) of **10**, 11.8 mmol of metal chloride, 0.1 g of DBU, and 20 mL of hexanol as solvent was refluxed for 600 min at 155 ºC and then filtered; **3-Fe** (yield: 10%) Found: C, 71.18%; H, 8.66%; N, 7.49%. Calcd. for C_88_H_128_N_8_O_8_Fe: C, 71.22%; H, 8.70%; N, 7.56%. IR (KBr): ν_max_/cm^-1^ 3300 (ν_C-H_), 2900 (ν_C-H_), 1590 (ν_C-O_), 1150 (ν_C-C_), 1090 (δ_C-H_), 720 (δ_C-H_). UV-Vis: λ_max_ Py/nm: 665. **3-Co** (yield: 10%) Found: C, 71.18%; H, 8.63%; N, 7.43%. Calcd. for C_88_H_128_N_8_O_8_Co: C, 71.17%; H, 8.68%; N, 7.54%. IR (KBr): νmax/cm^-1^ 3300 (ν_C-H_), 2900 (ν_C-H_), 1590 (ν_C-O_), 1150 (ν_C-C_), 1090 (δ_C-H_), 720 (δ_C-H_). UV-Vis: λ_max_ Py/nm: 665. **3-Cu** (yield: 10%) Found: C, 71.32%; H, 8.91%; N, 7.18%. Calcd. for C_88_H_128_N_8_O_8_Cu: C, 70.95%; H, 8.66%; N, 7.52%. IR (KBr): ν_max_/cm^-1^ 3300 (ν_C-H_), 2900 (ν_C-H_), 1590 (ν_C-O_), 1150 (ν_C-C_), 1090 (δ_C-H_), 720 (δ_C-H_). UV-Vis: λmax Py/nm: 665. **3-Zn** (yield: 10%) Found: C, 71.11%; H, 8.53%; N, 7.50%. Calcd. for C_88_H_128_N_8_O_8_Zn: C, 70.87%; H, 8.65%; N, 7.51%. IR (KBr): ν_max_/cm^-1^ 3300 (ν_C-H_), 2900 (ν_C-H_), 1590 (ν_C-O_), 1150 (ν_C-C_), 1090 (δ_C-H_), 720 (δ_C-H_). UV-Vis: λ_max_ Py/nm: 665.

### 3.6. Synthesis of Anthraquinocyanines ***4-M*** (M= Fe, Co, Zn)

Compounds **4** are a new type of phthalocyanine derivatives which were synthesized from a metal chloride, urea and 9,10-anthraquinone-2,3-dicarboxylic acid (**13**), which was synthesized from *o*-xylene and phthalic anhydride via o-(3,4-dimethybenzoyl)benzoic acid (**11)** and 2,3-dimethyl-9,10-anthraquinone (**12**) (Scheme 4).

#### 3.6.1. Synthesis of o-(3,4-dimethybenzoyl)benzoic acid (**11**)

Into a mixture of 16.3 g (0.11 mol) of phthalic dianhydride and 14 g of anhydrous aluminium chloride, 10.7 g of *o*-xylene in 25 mL of chloroform was added. The reaction mixture was refluxed for 60 min and the liquor then poured into a mixture of 30 g ice and 20 mL concentrated HCl, and then filtered. The product was recrystallized from diethyl ether; yield 81%. mp 170.6 °C. Found: C, 75.03%; H, 5.49%;. Calc for C_16_H_14_O_3_: C, 75.57%; H, 5.55 %. IR (KBr): ν_max_/cm^-1^ 3450(ν_O-H_), 2950 (ν_C-H_), 1760 (ν_C-O_), 1680 (ν_C-O_), 1601 (ν_C-C_), 1400 (ν_C-C_), 1225 (ν_C-C_), 1100 (ν_C-C_), 900(δ_C-H_), 710(δ_C-H_). ^1^H-NMR (CDCl_3_): δ, ppm 2.27 (s, 6H), 7.68 (m, 7H). MS *m/z* = 254.

#### 3.6.2. Synthesis of 2,3-dimethyl-9,10-anthraquinone (**12**)

Eighteen g of **11** and 2 mL of concentrated sulfuric acid were heated to 150 °C, then cooled and the crude product was recrystallized from benzene; yield 35%. mp 212.5 °C. Found: C, 80.89%; H, 5.02%; Calc for C_16_H_12_O_3_: C, 81.33%; H, 5.12 %. IR (KBr): ν_max_/cm^-1^ 2900 (ν_C-H_), 1760 (ν_C-O_), 1675 (ν_C-O_), 1420 (ν_C-C_), 1300 (ν_C-C_), 780(δC-H), 710(δC-H). ^1^H- NMR (CHCl_3_): δ, ppm 2.22 (s, 6H ), 7.46 (m, 7H). MS *m/z* = 236.

#### 3.6.3. Synthesis of 9,10-anthraquinone-2,3-dicarboxylic acid (**13**)

A solution of 4g of **12** in 4.36 mL concentrated sulfuric acid was added to 160 g of water, and 12 g of potassium permanganate (KMnO_4_) was added gradually to the solution. Reaction was allowed to continue at 90–95 °C for 10 min with stirring; excess KMnO_4_ was then removed with oxalic acid. The crude product was filtered and washed with hot water. The product dissolved in hot dilute ammonia solution, unreacted **12** being filtered off. Product **13** was precipitated from the filtrate by addition of HCl. The material was filtered, washed with hot water and dried; yield 66%. mp 325.0 °C. Found: C, 64.26%; H, 5.03%;. Calc for C_16_H_8_O_6_: C, 64.87%; H, 4.72 %. IR (KBr): ν_max_/cm^-1^ 3300 (ν_O-H_), 2900 (ν_C-H_), 1690 (ν_C-O_), 1600 (ν_C-C_), 1400 (ν_C-C_), 1260 (ν_C-C_), 980(δ_C-H_), 780 (δ_C-H_). ^1^H-NMR (CDCl_3_): δ, ppm 7.27 (s, 6H).

#### 3.6.4. Synthesis of compounds **4-M**

Compounds **4** were synthesized by the following procedure: a mixture of 1.00 g (3.30 mmol) of **13,** 2 g (34.0 mmol) of urea, 6.75 mmol of metal chloride, and 0.1 g of DBU was heated to 210 ºC until the reaction mixture was fused. The reaction product was washed with water and was dried; **4-Fe** (20%) Found: C, 70.60%; H, 2.22%; N, 10.29%. Calcd. for C_64_H_24_N_8_O_8_Fe: C, 70.96%; H, 2.28%; N, 10.13%. IR (KBr): ν_max_/cm^-1^ 3300 (ν_C-H_), 2900 (ν_C-H_), 1690 (ν_C-O_), 1470 (ν_C-C_), 980 (δ_C-H_), 780 (δ_C-H_). UV-Vis: λ_max_ Py/nm: 688. **4-Co** (23%) Found: C, 70.46%; H, 2.22%; N, 10.27%. Calcd. for C_64_H_24_N_8_O_8_Co: C, 70.17%; H, 2.26%; N, 10.30%. IR (KBr): ν_max_/cm^-1^ 3300(ν_C-H_), 2900 (ν_C-H_), 1690 (ν_C-O_), 1470 (ν_C-C_), 980 (δ_C-H_), 780 (δ_C-H_). UV-Vis: λ_max_ Py/nm: 688. **4-Zn** (22%) Found: C, 69.84%; H, 2.37%; N, 10.48%. Calcd. for C_64_H_24_N_8_O_8_Zn: C, 69.98%; H, 2.20%; N, 10.20%. IR (KBr): ν_max_/cm^-1^ 3300 (ν_C-H_), 2900 (ν_C-H_), 1690 (ν_C-O_), 1470 (ν_C-C_), 980 (δ_C-H_), 780 (δ_C-H_). UV-Vis: λ_max_ Py/nm: 688.

### 3.7. Synthesis of Alkylbenzopyridoporphyrazineporphyrazines ***5***

In the synthesis of **5**, a mixture of 3,6-didecylphthalonitrile and 3,4-pyridinecarbodinitrile was dissolved in pentanol (7 mL) and zinc chloride (0.05 g) was added (Scheme 5). Zinc 1,4-didecylbenzo-tris(3,4-pyrido)porphyrazine (**5b**), zinc bis(1,4-didecylbenzo)-3,4-pyridoporphyrazine (**5c**) and zinc tris(1,4-didecylbenzo)-3,4-pyridoporphyrazine (**5d**) were synthesized from mixtures of 3,6-didecylphthalonitrile and 3,4-dicyanopyridine in 3 (0.18 g, 0.30 mmol):1 (0.02 g, 0.15 mmol), 1 (0.12 g, 0.29 mmol):1 (0.04 g, 0.29 mmol) and 1 (0.06 g, 0.15 mmol):3 (0.06 g, 0.45 mmol) ratios, respectively. Zinc tetra-3,4-pyridoporphyrazine (**5a**) and zinc octadecylphthalocyanine (**5e**) were synthesized from 3,4-dicyanopyridine [28,54] and 3,6-didecylphthalonitrile [33,59], respectively.

The mixtures were heated for 240 min in the presence of DBU as a catalyst. After cooling, the reaction mixture was dissolved in toluene (50 mL) and the solution filtered. The solvent was removed by evaporation. The products were separated and purified by TLC (eluent: toluene). **5a** (29%) Found: C, 57.81%; H, 2.11%; N, 28.90%. Calcd. for C_28_H_12_N_12_Zn: C, 57.79%; H, 2.08%; N, 28.89%. IR (KBr): ν_max_/cm^-1^ 2960 (ν_C-H_), 1500 (ν_C-C_), 1470 (ν_C-C_), 1450 (ν_C-C_), 1210 (δ_C-H_), 1090 (δ_C-H_), 790 (δ_C-H_). UV-Vis: λ_max_ Py/nm: 675; **5b** (65%) Found: C, 68.31%; H, 6.22%; N, 17.90%. Calcd. for C_49_H_53_N_11_Zn: C, 68.32%; H, 6.22%; N, 17.89%. IR (KBr): ν_max_/cm^-1^ 2960 (ν_C-H_), 1600 (ν_C-C_), 1460 (ν_C-C_), 1440 (ν_C-C_), 1210 (δ_C-H_), 1080 (δ_C-H_), 720 (δ_C-H_). ^1^H-NMR (90 MHz, CDCl_3_): δ, ppm 7.4 (11.0H), 2.8 (4.2H), 1.3 (33.4H), 0.9 (6.7H). UV-Vis: λ_max_ toluene/nm: 686. **5c** (84%) Found: C, 73.67%; H, 8.30%; N, 12.28%. Calcd. for C_70_H_94_N_10_Zn: C, 73.68%; H, 8.30%; N, 12.28%. IR (KBr): ν_max_/cm^-1^ 2970 (ν_C-H_), 1600 (ν_C-C_), 1500 (ν_C-C_), 1470 (ν_C-C_), 1450 (ν_C-C_), 1210 (δ_C-H_), 1090 (δ_C-H_), 720 (δ_C-H_). ^1^H-NMR (90 MHz, CDCl_3_): δ, ppm 7.4 (10.0H), 2.8 (8H), 1.3 (63.8H), 0.9 (12.3H). UV-Vis: λ_max_ toluene/nm: 686. **5d** (84%) Found: C, 76.94%; H, 9.57%; N, 8.86%. Calcd. for C_91_H_135_N_9_Zn: C, 76.94%; H, 9.58%; N, 8.88%. IR (KBr): ν_max_/cm^-1^ 2970 (ν_C-H_), 1600 (ν_C-C_), 1500 (ν_C-C_), 1470 (ν_C-C_), 1210 (δ_C-H_), 1100 (δ_C-H_), 720 (δ_C-H_). ^1^H-NMR (90 MHz, CDCl_3_): δ, ppm 7.4 (9.0H), 2.8 (12.3H), 1.3 (95.9H), 0.9 (18.8H). UV-Vis: λ_max_ toluene/nm: 686. **5e** (96%) Found: C, 79.12%; H, 10.41%; N, 6.61%. Calcd. for C_112_H_176_N_8_Zn: C, 79.12%; H, 10.43%; N, 6.59%. IR (KBr): ν_max_/cm^-1^ 2970 (ν_C-H_), 1600 (ν_C-C_), 1500 (ν_C-C_), 1470 (ν_C-C_), 1210 (δ_C-H_), 1100 (δ_C-H_), 720 (δ_C-H_). ^1^H-NMR (90 MHz, CDCl_3_): δ, ppm 7.4 (8.0H), 2.8 (16.0H), 1.3 (128.0H), 0.9 (24.1H). UV-Vis: λ_max_ toluene/nm: 703.

The regioisomers of **5c** were separated by TLC (Merk Silica gel 60 F_254_ on aluminium sheet, eluent: toluene - Py, 7:3) into four green- to blue-colored fractions. These fractions were numbered from 1, 2, 3 and 4, according to their R_f_ values of 0.95, 0.91, 0.75 and 0.65, respectively. Each fraction was recovered by scraping from the TLC plate, dissolved in pyridine, the solution filtered, and the solvent removed. **Fraction 1**: ^1^H-NMR (benzene-d_6_); δ ppm 0.93 (t, 12H), 1.21-1.88 (m, 48H), 1.88-2.23 (m, 8H), 2.29-2.69 (m, 8H), 4.13 (t, 4H), 4.32 (t, 4H), 7.35 (d, 2H), 7.49 (d, 2H), 8.30 (m, 6H). **Fraction 2**: ^1^H-NMR (benzene-d_6_); δ ppm 0.92 (t, 12H), 1.24-1.80 (m, 48H), 1.82-2.29 (m, 8H), 2.32-2.62 (m, 8H), 4.12 (t, 4H), 4.30 (t, 4H), 7.36 (d, 2H), 7.49 (d, 2H), 8.31 (m, 6H). **Fraction 3**: ^1^H-NMR (benzene-d_6_); δ ppm 0.92 (t, 12H), 1.16-1.95 (m, 48H), 1.95-2.20 (m, 4H), 2.20-2.36 (m, 4H), 2.36-2.70 (m, 8H), 3.99 (t, 4H), 4.23 (t, 4H), 7.36 (d, 2H), 7.49 (d, 2H), 8.30 (m, 6H). **Fraction 4**: ^1^H-NMR (benzene-d_6_); δ ppm 0.92 (t, 12H), 1.27-1.83 (m, 48H), 1.83-2.26 (m, 8H), 2.30-2.68 (m, 8H), 4.07 (t, 4H), 4.29 (t, 4H), 7.35 (d, 2H), 7.48 (d, 2H), 8.31 (m, 6H).

Quaternization of **5c** was performed as follows: zinc **5c** (0.17 g, 0.15 mmol) was reacted with quaternizing agents such as MCAA (0.57 g, 6 mmol), DES (0.1 g, 0.6 mmol) and DMS (0.2 g, 1.5 mmol), respectively, in DMF as solvent at 140°C for 120 min. The reaction mixture was dissolved in acetone (20 mL), cooled to room temperature and the solution filtered. The solvent was removed. The products were purified by TLC (eluent: THF-toluene, 8:2). Each product was recovered by scraping from the TLC plate, dissolving in Py, filtering the solution, and removal of the solvent. **5c with MCAA**: dark blue solid (32 mg; yield 23%). ^1^H-NMR (DMSO-d_6_); δ ppm 0.85 (m, 12H, CH_3_), 1.19-1.71 (m, 48H, γ-CH_2_), 1.79-2.12 (m, 8H, β-CH_2_), 2.27-2.68 (m, 8H, β-CH_2_), 4.15 (m, 4H, α-CH_2_), 4.39 (m, 4H, α-CH_2_), 6.19 (s, 2H, CH_2_), 7.37 (m, 4H, arm), 8.32 (m, 6H, Py). IR (KBr): ν_max_/cm^-1^ 3050 (ν_C-H_), 2980 (ν_C-H_), 1730 (ν_C-O_), 1620 (ν_C-C_), 1400 (ν_C-C_), 1210 (δ_C-H_), 1080 (δ_C-H_), 790 (δ_C-H_), 690 (δ_C-H_). UV-Vis (λ_max_toluene/nm) 686: (λ_max_Py/nm) 690: (λ_max_water/nm) 681; Found: C, 45.02%; H, 2.49%; N, 17.48%. Calcd. for C_74_H_100_N_10_O_4_Zn: C, 45.05%; H, 2.52%; N.17.50%. **5c with DES**: blue solid (37 mg, yield 17%). ^1^H-NMR (DMSO-d_6_) δ, ppm 0.86 (m, 12H, CH_3_), 1.02-1.70 (m, 48H, γ-CH_2_), 1.88-2.11 (m, 8H, β-CH_2_), 2.30-2.68 (m, 8H, β-CH_3_), 4.11 (m, 4H, α-CH_2_), 4.25 (m, 4H, α-CH_2_), 7.38 (m, 4H, arom), 8.18 (m, 4H, Py). IR (KBr) ν_max_/cm^-1^ 3050 (ν_C-H_), 2960 (ν_C-H_), 1500 (ν_C-C_), 1450 (ν_C-C_), 1400 (ν_C-C_), 1340 (ν_S-O_), 1180 (ν_S-O_), 1250 (δ_C-H_), 920 (δ_C-H_), 760 (δ_C-H_), 590 (δ_C-S_). UV-Vis (λ_max_toluene/nm) 687: (λ_max_Py/nm) 731: (λ_max_water/nm) 679. Found: C,37.10%; H, 1.78%; N. 18.49%. Calcd. for C_70_H_98_N_10_S_2_O_6_Zn: C, 37.11%; H, 1.78%; N, 18.54%. **5c with DMS**: dark blue solid (53 mg, yield 28%). ^1^H-NMR (DMSO-d_6_): δ ppm 0.88 (m, 12H, CH_3_), 1.14-1.72 (m, 48H, γ-CH_2_), 1.82-2.17 (m, 8H, β-CH_2_), 2.29-2.62 (m, 8H, β-CH_2_), 4.06 (s, 6H, CH_3_), 4.27 (m, 4H, α-CH_2_), 4.51 (m, 4H, α-CH_2_), 7.34 (m, 4H, arom), 8.23 (m, 6H, Py); IR (KBr): ν_max_/cm^-1^ 3060 (ν_C-H_), 2980 (ν_C-H_), 1500 (ν_C-C_), 1450 (ν_C-C_), 1400 (ν_C-C_), 1250 (δ_C-H_), 1100 (δ_C-H_), 950 (δ_C-H_), 830 (δ_C-H_), 660 (δ_C-H_). UV-Vis (λ_max_toluene/nm) 739: (λ_max_Py/nm) 739: (λ_max_water/nm) 684. Found: C, 49.03%; H, 3.08%; N, 21.40%. Calcd. for C_72_H_100_N_10_Zn: C, 49.03%; H, 3.09%; N, 21.43%.

Quaternized regioisomers of **5c**: Regioisomers of **5c** (0.4 mg, 0.35 μmol) were reacted with a quaternizing agent such as DMS (0.5 mg, 3.5 μmol) in DMF as solvent at 140 °C for 120 min. The products were purified with TLC (eluent: THF-toluene, 8: 2). Each product was recovered by scraping from the TLC plate, dissolving in pyridine, filtering the solution and removal of the solvent. **Fraction 1 with DMS**: dark blue solid (0.053 mg, yield 13 %). ^1^H-NMR (DMSO-d_6_): δ ppm 0.86 (t, 12H, CH_3_), 1.15-1.70 (m, 48H, γ-CH_2_), 1.82-2.12 (m, 8H, β-CH_2_), 2.19-2.49 (m, 8H, β-CH_2_), 3.67 (s, 6H, CH_3_), 3.92 (t, 4H, α-CH_2_), 4.19 (t, 4H, α-CH_2_), 7.37 (m, 4H, arom), 8.20 (m, 6H, Py); IR (KBr): ν_max_/cm^-1^) 3070 (ν_C-H_), 2980 (ν_C-H_), 1500 (ν_C-C_), 1400 (ν_C-C_), 1250 (δ_C-H_), 1100 (δ_C-H_), 950 (δ_C-H_), 820 (δ_C-H_), 660 (δ_C-H_); UV-Vis (λ_max_ toluene/nm) 680: (λ_max_ Py/nm) 703: (λ_max_ water/nm) 690; Fluorescence (F_max_ Py/nm) 687: (F_max_ water/nm) 695. Found: C, 49.00%; H, 3.01%; N, 21.33%. Calcd. for C_72_H_100_N_10_Zn: C, 49.03%; H, 3.09%; N, 21.43%. **Fraction 2 with DMS**: dark blue solid (0.065 mg, yield 15 %). ^1^H-NMR (DMSO-d_6_): δ ppm 0.87 (t, 12H, CH_3_), 1.16-1.73 (m, 48H, γ-CH_2_), 1.73-2.10 (m, 8H, β-CH_2_), 2.10-2.55 (m, 8H, β-CH_2_), 3.90 (s, 6H, CH_3_), 3.99 (t, 4H, α-CH_2_), 4.17 (t, 4H, α-CH_2_), 7.23 (m, 4H, arom), 8.25 (m, 6H, Py). IR (KBr): ν_max_/cm^-1^ 3070 (ν_C-H_), 2980 (ν_C-H_), 1500 (ν_C-C_), 1400 (ν_C-C_), 1250 (δ_C-H_), 1100 (δ_C-H_), 980 (δ_C-H_), 830 (δ_C-H_), 660 (δ_C-H_); UV-Vis (λ_max_ toluene/nm) 689: (λ_max_ Py/nm) 675: (λ_max_ water/nm) 686; Fluorescence (F_max_ Py/nm) 684: (F_max_ water/nm) 690; Found: C, 48.99%; H, 2.98%; N, 21.22%. Calcd. for C_72_H_100_N_10_Zn: C, 49.03%; H, 3.09%; N, 21.43%. **Fraction 3 with DMS**: dark blue solid (0.055 mg, yield 11 %). ^1^H-NMR (DMSO-d_6_): δ ppm 0.85 (m, 12H, CH_3_), 1.09-1.63 (m, 48H, γ-CH_2_), 1.70-2.11 (m, 8H, β-CH_2_), 2.11-2.42 (m, 8H, β-CH_2_), 3.86 (s, 6H, CH_3_), 3.97 (t, 4H, α-CH_2_), 4.21 (t, 4H, α-CH_2_), 7.23-7.33 (m, 4H, arom), 8.21 (m, 6H, Py). IR (KBr): λ_max_/cm^-1^ 3080 (ν_C-H_), 2970 (ν_C-H_), 1500 (ν_C-C_), 1400 (ν_C-C_), 1250 (δ_C-H_), 1100 (δ_C-H_), 950 (δ_C-H_), 830 (δ_C-H_), 660 (δ_C-H_). UV-Vis (λ_max_ toluene/nm) 681: (λ_max_ Py/nm) 689: (λ_max_ water/nm) 689; Fluorescence (F_max_ Py / nm) 690: (F_max_ water / nm) 698; Found: C, 49.03%; H, 3.03%; N, 21.40%. Calcd. for C_72_H_100_N_10_Zn: C, 49.03%; H, 3.09%; N, 21.43%. **Fraction 4 with DMS**: dark blue solid (0.078 mg, yield 19 %). ^1^H-NMR (DMSO-d_6_): δ ppm 0.87 (t, 12H, CH_3_), 1.20-1.63 (m, 48H, γ-CH_2_), 1.71-2.17 (m, 8H, β-CH_2_), 2.17-2.47 (m, 8H, β-CH_2_), 3.85 (s, 6H, CH_3_), 4.09 (t, 4H, α-CH_2_), 4.27 (t, 4H, α-CH_2_), 7.21-7.36 (m, 4H, arom), 8.22 (m, 6H, Py). IR (KBr): λ_max_/cm^-1^ 3070 (ν_C-H_), 2980 (ν_C-H_), 1500 (ν_C-C_), 1400 (ν_C-C_), 1250 (δ_C-H_), 1100 (δ_C-H_), 950 (δ_C-H_), 830 (δ_C-H_), 660 (δ_C-H_); UV-Vis (λ_max_ toluene/nm) 677: (λ_max_ Py/nm) 730: (λ_max_ water/nm) 673; Fluorescence (F_max_ Py/nm) 686: (F_max_ water/nm) 687; Found: C.49.02: H. 3.08: N. 21.43. Calcd. for C_72_H_100_N_10_Zn: C. 49.03: H. 3.09: N.21.43.

### 3.8. Synthesis of subphthalocyanine derivatives ***6***

Compound **6a** was synthesized from 1,2-dicyanobenzene. Compound **6c** was synthesized from 1,2-dicyano-3,4,5,6-tetrafluorobenzene. Compounds **6d-g** were synthesized from the precursors, 1,2-dicyano-3,6-bis(thiobutyl)-4,5-difluorobenzene (**14a**) [36], 1,2-dicyano-3,4,5,6-tetrakis(thiobutyl)-benzene (**14b**) [36], 1,2-dicyano-3,6-bis(thiophenyl)-4,5-difluorobenzene (**14c**) [36] and 1,2-dicyano-3,4,5,6-tetrakis(thiophenyl)benzene (**14d**) [36], respectively.

Compound **6a**: A solution of boron trichloride (20.5 mL, 0.02 mol, 1M in hexane) was added into a mixture of 1,2-benzenedicarbonitrile (5.0 g, 0.04 mol) and 1-chloronaphtahalene (12 mL) under an argon atmosphere at -3°C. After addition, the reaction mixture was heated to 120 °C with stirring. Hexane was distilled off. It was heated to 250°C for 10 minutes and cooled to room temperature. The product was extracted with petroleum ether for 1440 min and then toluene for 120 min on a Soxhlet extractor. The obtained product was recrystallized from ethanol (Scheme 6).

SubPC derivatives **6b-g** were synthesized in accordance with above-mentioned SubPC synthetic method. **6a** (71%) Found: C, 66.88%; H, 2.81%; N, 19.50%. Calcd. for C_24_H_12_N_6_BCl: C, 66.88%; H, 2.81%; N, 19.59%. IR (KBr): ν_max_/cm^-1^ 3200 (ν_C-H_), 3070 (ν_C-H_), 2370 (ν_C-N_), 1611 (ν_C-C_), 1452 (ν_C-C_), 1304 (ν_C-H_), 1130 (δ_C-H_), 950 (δ_C-S_). ^1^H-NMR (CDCl_3_): δ, ppm 7.21 (s, 12H). MS *m/z* = 431. UV-Vis: λ_max_ (toluene)/nm: 564. **6b** (39%) Found: C, 72.18%; H, 6.06%; N, 14.01%. Calcd. for C_36_H_36_N_6_BCl: C, 72.18%; H, 6.06%; N, 14.01%. IR (KBr): ν_max_/cm^-1^ 2980 (ν_C-H_), 1720 (ν_C-C_), 1620 (ν_C-C_), 1450 (ν_C-H_), 1400 (ν_C-H_), 1360 (δ_C-H_), 1280 (δ_C-H_), 1190 (δC-H), 760 (δ_C-H_). ^1^H-NMR (CDCl_3_): δ, ppm 7.80 (s, 3H), 7.49 (s, 3H), 7.32 (s, 3H), 1.35 (s, 9H). MS *m/z* = 599. UV-Vis: λ_max_ toluene/nm: 566. **6c** (26%) Found: C, 50.39%; N, 14.70%. Calcd. for C_24_N_6_BClF_12_: C, 50.39%; H, 0.00%; N, 14.70%. IR (KBr): ν_max_/cm^-1^ 1490 (ν_C-H_), 1450 (ν_C-N_), 1270 (ν_C-F_), 1220 (ν_C-F_), 1120 (ν_C-H_), 960 (δ_C-H_), 800 (δ_C-H_), 760 (δ_C-S_). MS *m/z* = 647. UV-Vis: λ_max_ toluene/nm: 572. **6d** (8%) Found: C, 53.99%; H, 5.10%; N, 7.84%. Calcd. for C_48_H_54_N_6_BClF_6_S_6_: C, 53.99%; H, 5.10%; N, 7.87%. IR (KBr): ν_max_/cm^-1^ 2980 (ν_C-H_), 2150 (ν_C-N_), 1600 (ν_C-C_), 1500 (ν_C-C_), 1450 (ν_C-C_), 1150 (δ_C-H_), 850 (δ_C-H_). ^1^H-NMR (CDCl_3_): δ, ppm 3.15 (t, 12H), 1.51 (m, 24H), 0.90 (t,18H). MS *m/z* = 1068. UV-Vis: λ_max_ (toluene)/nm: 596. **6e** (5%) Found: C, 60.69%; H, 2.55%; N, 7.08%. Calcd. for C_60_H_30_N_6_BClF_6_S_6_: C, 60.66%; H, 2.55%; N, 7.08%. IR (KBr): ν_max_/cm^-1^ 3050 (ν_C-H_), 2250 (ν_C-N_), 1580 (ν_C-C_), 1450 (ν_C-C_), 1250 (ν_C-F_), 1090 (δ_C-H_), 670 (δ_C-S_). ^1^H-NMR (CDCl_3_): δ, ppm 7.26 (m, 30H). MS *m/z* = 1188. UV-Vis: λ_max_ toluene/nm: 621. **6f** (17%) Found: C, 58.07%; H, 7.31%; N, 5.65%. Calcd. for C_72_H_108_N_6_BClS_12_: C, 58.07%; H, 7.31%; N, 5.65%. IR (KBr): ν_max_/cm^-1^ 2980 (ν_C-H_), 2125 (ν_C-N_), 1580 (ν_C-C_), 1500 (ν_C-C_), 1460 (ν_C-C_), 1000 (ν_C-H_), 800 (δ_C-H_). ^1^H-NMR (CDCl_3_): δ, ppm 3.16 (t, 24H), 1.50 (m, 48H), 0.91 (t, 36H). UV-Vis: λ_max_ toluene/nm: 612. **6g** (13%) Found: C, 66.67%; H, 3.50%; N, 4.86%. Calcd. for C_96_H_60_N_6_BClS_12_: C, 66.67%; H, 3.50%; N, 4.86%. IR (KBr): ν_max_/cm^-1^ 3080 (ν_C-H_), 2190 (ν_C-N_), 1600 (ν_C-C_), 1500 (ν_C-C_), 1460 (ν_C-C_), 1000 (δ_C-H_), 800 (δ_C-H_). ^1^H-NMR (CDCl_3_): δ, ppm 7.26 (m, 60H). UV-Vis: λ_max_ (toluene)/nm: 634.

### 3.9. Synthesis of octakis(thiophenyl)phthalocyanines ***15***

The target compounds **15** were synthesized in three steps via intermediates **16** and **17a-c**. Intermediate **16** was synthesized by the following procedure: 2,3-dicyanohydroquinone (4.80 g, 30 mmol) in dichloromethane (100 mL) and Py (5.93 g, 75 mmol) was treated with trifluoromethanesulfonic anhydride (21.16 g, 75 mmol) under nitrogen at -78 °C. After the reaction, the mixture was allowed to warm slowly to room temperature; stirring was continued for 1,440 min. The mixture was poured into water (600 mL) and the organic layer was extracted using dichloromethane (5 × 100 mL). The extract was washed in turn with water, 2%-hydrochloric acid, water, brine and water, and dried on magnesium sulfate (MgSO_4_). The filtrate and the solvent evaporated. The crude product was recrystallized from dichloromethane to afford **16** (6.35 g, 50%) as colorless needles. Found: C, 28.32%; H, 0.48%; N, 6.59%. Calcd. for C_10_H_2_F_6_N_2_S_2_O_6_: C, 28.31%; H, 0.48%; F, 26.87%; N, 6.60%; O, 22.63%; S, 15.12%. IR (KBr): cm-1 3115 (νC-H), 2550 (νC-N), 1601 (νC-C), 1472 (νC-C), 1439 (νC-C), 1134 (νS=O). ^1^H-NMR (400 MHz, DMSO-d_6_): δ, ppm 8.44 (s, 2H).

Intermediates **17a-c** were synthesized as follows: to a mixture of **16** (0.85 g, 2 mmol), potassium carbonate (1.16 g) and DMSO (15 mL), thiophenols such as *p*-toluenethiol, 4-methoxybenzenethiol and *ter*t-butylthiophenol (4 mmol) were added; the mixture was reacted at room temperature for 1,440 min in nitrogen atmosphere. The reaction products were poured into water (300 mL), and the organic layer extracted using dichloromethane (5 × 100 mL), and dried on MgSO_4_. The filtrate and the solvent evaporated. The crude product was washed with methanol (3 × 50 cm^3^) and recrystallized from toluene to afford **17** as a yellow solid. 3,6-bis(thiophenylmethyl)phthalonitrile (**2a**) (0.27 g, 35%) Found: C, 70.90%; H, 4.30%; N, 7.50%. Calcd. for C_22_H_16_N_2_S_2_: C, 70.93%; H, 4.33%; N, 7.52%; S, 17.22. IR (KBr): cm^-1^ 3050 (ν_C-H_), 2970 (ν_C-H_), 2218 (ν_C-N_), 1600 (ν_C-C_), 1535 (ν_C-C_), 1490 (ν_C-C_), 1435 (ν_C-C_), 1210, 809 (δ_C-H_). ^1^H-NMR (DMSO-*d*_6_): δ, ppm 7.54 (d, 4H), 7.46 (d, 4H), 7.35 (s2H), 2.66 (tt, 6H); 3,6-bis(thiophenylmethoxy)phthalonitrile (**2b**) (0.28 g, 34%) Found: C, 65.30%; H, 4.00%; N, 6.93%. Calcd. for C_22_H_16_N_2_S_2_O_2_: C, 65.32%; H, 3.99%; N, 6.93%; S, 15.82%; O, 7.91%. IR (KBr): cm^-1^ 3050 (ν_C-H_), 2970 (ν_C-H_), 2216 (ν_C-N_), 1600 (ν_C-C_), 1540 (ν_C-C_), 1487 (ν_C-C_), 1430 (ν_C-C_), 1210, 810 (δ_C-H_). ^1^H-NMR (DMSO-*d*_6_): δ, ppm 7.49 (d, 4H), 7.06 (d, 4H), 7.04 (s2H), 3.79 (s, 6H); 3,6-bis(thiophenyl *ter*t-butyl)phthalonitrile (**2c**) (0.38g, 42%) Found: C, 73.65%; H, 6.18%; N, 6.11%. Calcd. for C_28_H_28_N_2_S_2_: C, 73.64%; H, 6.18%; N, 6.13%; S, 14.04%. IR (KBr): cm^-1^ 3040 (ν_C-H_), 2960 (ν_C-H_), 2210 (ν_C-N_), 1600 (ν_C-C_), 1500 (ν_C-C_), 1460 (ν_C-C_), 1210, 808 (δ_C-H_). ^1^H-NMR (DMSO-*d*_6_): δ, ppm 7.50 (d, 4H), 7.46 (d, 4H), 7.26 (s, 2H), 1.28 (s, 18H).

The target compounds **15** were synthesized following procedure; A solution of **17** (0.25 mmol), the appropriate metal chloride or metal acetate (0.065 mmol), DBU (0.2 mL) as a catalyst and 1-pentanol (10 mL) was refluxed for 420 min. After cooling to room temperature, the reaction products were poured into methanol to form a precipitate, which was washed with water and methanol, and chromatographed on silica gel with toluene as eluent. The 1,4,8,11,15,18,22,25-octakis(thiophenyl-methyl)phthalocyanines (**15a**); **15a-Cu** (0.02 g, 18%) Found: C, 67.98%; H, 4.51%; N, 7.01%. Calcd. for C_88_H_65_N_8_S_8_Cu: C, 67.99%; H, 4.21%; N, 7.21%; S, 16.50%; Cu, 4.09%. MS (FAB): *m/z* found 1554, Calcd. 1554.59: **15a-Co** (0.02 g, 24%) Found: C, 68.10%; H, 4.20%; N, 7.25%. Calcd. for C_88_H_65_N_8_S_8_Co: C, 68.19%; H, 4.23%; N, 7.23%; S, 16.55%; Co, 3.80%. MS (FAB): *m/z* found 1550, Calcd. 1549.97: **15a-Ni** (0.03 g, 28%) Found: C, 68.18%; H, 4.20%; N, 7.13%. Calcd. for C_88_H_65_N_8_S_8_Ni: C, 68.20%; H, 4.23%; N, 7.23%; S, 16.55%; Ni, 3.79%. MS (FAB): *m/z* found 1550, Calcd. 1549.73: **15a-Zn** (0.02 g, 24%) Found: C, 67.87%; H, 4.23%; N, 7.11%. Calcd. for C_88_H_65_N_8_S_8_Zn: C, 67.91%; H, 4.21%; N, 7.20%; S, 16.48%; Zn, 4.20%. MS (FAB): *m/z* found 1556, Calcd. 1556.43: **15a-Pb** (0.04 g, 34%) Found: C, 62.29%; H, 3.88%; N, 6.40%. Calcd. for C_88_H_65_N_8_S_8_Pb: C, 62.24%; H, 3.86%; N, 6.60%, S, 15.11%; Pb, 12.19%. MS (FAB): *m/z* found 1699, Calcd. 1698.24: **15a-H_2_** (0.04 g, 19%) Found: C, 70.85%; H, 4.66%; N, 7.51%. Calcd. for C_88_H_66_N_8_S_8_: C, 70.84%; H, 4.46%; N, 7.51%, S, 17.1%. MS (FAB): *m/z* found 1492, Calcd. 1492.04; 1,4,8,11,15,18,22,25-octakis(thiophenylmethoxy)phthalocyanines (**15b**); **15b-Cu** (0.03 g, 27%) Found: C, 62.88%; H, 3.91%; N, 6.65%. Calcd. for C_88_H_65_N_8_S_8_ O_8_Cu: C, 62.82%; H, 3.89%; N, 6.66%; S, 15.25%; O, 7.61%; Cu, 3.77%. MS (FAB): *m/z* found 1682, Calcd. 1682.58: **15b-Co** (0.02g, 21%) Found: C, 63.00%; H, 3.89%; N, 6.59%. Calcd. for C_88_H_65_N_8_S_8_O_8_Co: C, 62.99%; H, 3.90%; N, 6.68%; S, 15.29%; O, 7.63%; Co, 3.51%. MS (FAB): *m/z* found 1678, Calcd. 1677.97: **15b-Ni** (0.03 g, 25%) Found: C, 63.01%; H, 3.92%; N, 6.67%. Calcd. for C_88_H_65_N_8_S_8_O_8_Ni: C, 63.01%; H, 3.92%; N, 6.67%; S, 15.29%; O, 7.63%; Ni, 3.49%. MS (FAB): *m/z* found 1678, Calcd. 1677.73: **15b-Zn** (0.03 g, 29%) Found: C, 62.75%; H, 3.95%; N, 6.65%. Calcd. for C_88_H_65_N_8_S_8_O_8_Zn: C, 62.75%; H, 3.89%; N, 6.65%; S, 15.23%; O, 7.60%; Zn, 3.88%. MS (FAB): *m/z* found 1684, Calcd. 1684.42: **15b-Pb** (0.04 g, 35%) Found: C, 57.88%; H, 3.59%; N, 6.15%. Calcd. for C_88_H_65_N_8_S_8_O_8_Pb: C, 57.88%; H, 3.59%; N, 6.14%; S, 14.05%; O, 7.01%; Pb, 11.33%. MS (FAB): *m/z* found 1827, Calcd. 1826.23: **15b-H_2_** (0.02 g, 8%) Found: C, 65.24%; H, 4.11%; N, 6.93%. Calcd. for C_88_H_66_N_8_O_8_S_8_: C, 65.24%; H, 4.11%; N, 6.92%, O, 7.90, S, 15.18%. MS (FAB): *m/z* found 1620, Calcd. 1620.03; 1,4,8,11,15,18,22,25-octakis-(thiophenyl *tert*-butyl)phthalocyanines (**15c**); **15c-Cu** (0.02 g, 20%) Found: C, 71.13%; H, 6.05%; N, 5.87%. Calcd. for C_112_H_113_N_8_S_8_Cu: C, 61.13%; H, 6.02%; N, 5.92%; S, 13.56%; Cu, 3.37%. MS (FAB): *m/z* found 1891, Calcd. 1891.22: **15c-Co** (0.02 g, 21%) Found: C, 71.24%; H, 6.04%; N, 5.93%. Calcd. for C_112_H_113_N_8_S_8_Co: C, 71.30%; H, 6.04%; N, 5.94%; S, 13.60%, Co, 3.12%. MS (FAB): *m/z* found 1886, Calcd. 1886.61: **15c-Ni** (0.03 g, 23%) Found: C, 71.31%; H, 6.04%; N, 5.89%. Calcd. for C_112_H_113_N_8_S_8_Ni: C, 71.31%; H, 6.04%; N, 5.94%; S, 13.60%; Ni, 3.11%. MS (FAB): *m/z* found 1886, Calcd. 1886.37: **15c-Zn** (0.03 g, 28%) Found: C, 71.00%; H, 6.01%; N, 5.92%. Calcd. for C_112_H_113_N_8_S_8_Zn: C, 71.06%; H, 6.02%; N, 5.92%; S, 13.55%; Zn, 3.45%. MS (FAB): *m/z* found 1893, Calcd. 1803.07: **15c-Pb** (0.04 g, 34%) Found: C, 66.12%; H, 5.63%; N, 5.53%. Calcd. for C_112_H_113_N_8_S_8_Pb: C, 66.11%; H, 5.60%; N, 5.51%; S, 12.61%; Pb, 10.17%. MS (FAB): *m/z* found 2035, Calcd. 2034.88: **15c-H_2_** (0.02 g, 9%) Found: C, 73.55%; H, 6.26%; N, 6.13%. Calcd. for C_112_H_114_N_8_S_8_: C, 73.56%; H, 6.28%; N, 6.13%, S, 14.03%. MS (FAB): *m/z* found 1829, Calcd. 1828.68.

### 3.10. Electrochemical measurements

CV of metal octakis(hexoxymethyl)phthalocyanines and metal anthraquinocyanine were carried out with a BAS CV-50W voltammetric analyzer at room temperature in DMSO containing a 0.10 mol·L^-1^ solution of TBAP in a stream of nitrogen. CVs were recorded by scanning the potential at the rate of 50 mV s^-1^. The working and counter electrodes were platinum wires and the reference electrode was a Ag/AgCl saturated sodium chloride electrode. The chronoamperometry was measured following an applied voltage pulse from -1200 to 0 mV vs. Ag/AgCl and from 0 to +1600 mV vs. Ag/AgCl, and the reversible pulse.

### 3.11. Laser- flash photolysis

Laser flash photolysis in film was performed using a total reflection sapphire cell (10 · 30 mm, 1 mm thick, and both of the short side were cut at a 45 degree angle), which was spin-coated with a 1.2 μm thick photopolymer film. An excitation light pulse (20 ms, 355 nm and 10 mJ per pulse) from a YAG laser was expanded and exposed over the entire sample cell. A monitoring light from a xenon lamp passed through the multireflection cell which was connected to the head of an optical fiber attached to a monochrometer equipped with a photomultiplier or to a spectral multichannel analyzer system. The films were prepared as follows: a 10 wt% PMMA solution was made up in cyclohexanone, and allylbenzopyridoporphyrazines were added to this solution by dissolving to a thickness of 1.2 μm thick by spin-coating a solution onto a sapphire cell. After that the films were covered with a PVA solution.

## 4. Conclusions

M-PCs **1**, **2**, **3**, and a new type of phthalocyanine derivative, **4** have been synthesized. Then, the cyclotetramerization products from a 1:1 mol mixture of 3,6-didecylphthalonitrile and 3,4-dicynopyridine, zinc bis(1,4-didecylbenzo)bis(3,4-pyrido)porphyrazines, **5** were synthesized. The cyclotetramerization products have position isomers. The isomers of **5c** was separated into fore fractions. Moreover, metal phthalocyanine analogous, SubPCs and its derivatives which possessed substituents such as thiobutyl and thiophenyl were synthesized.

Electrochemical measurements were performed above mentioned phthalocyanine derivatives and analogous in order to examine their electron transfer abilities and electrochemical mechanism in an organic solvent.

Photochemical properties such as the triplet state lifetime were measured using laser-flash photolysis. The triplet state of zinc non-peripheral substituted alkylbenzopyridoporphyrazines, **5c** and position isomers were measured, using laser-flash photolysis in a PMMA film. In generally, the triplet lifetime increased as the number of Py groups in the molecule increased. However, the lowest symmetric isomer having two Py rings in the molecule showed the longest triplet state lifetime. The compound was suitable for use as a photosensitizer in photodynamic cancer therapy.

Thiobutyl and thiophenyl substituted subPC derivatives **6** were measured sensitization properties. Sensitization properties of **6** with 2,4,6-tris(trichloromethyl)-1,3,5-triazine as radical generation reaction in a PMMA film have been investigated by laser-flash photolysis.

Phthalocyanines having S-aryl groups at non-peripheral position, i.e., 1,4,8,11,15,18,22,25-octakis(thio-phenylmethyl)phthalocyane compounds **15** can be synthesized. Their strong Q band absorption peaks appear around 800 nm. The synthesized phthalocyanines have high solubility in organic solvents, high heat resistance without discotic liquid crystal behavior, no absorptions in the visible region, and a simple structure without any regioisomers.
